# Ferroelectric Hafnium Oxide for In-Memory Computing: Advancing Devices, Circuit Architectures, and System-Level Integration

**DOI:** 10.3390/mi17080931

**Published:** 2026-08-04

**Authors:** Chengyu He, Wei Li, Jianjun Li, Qiquan Li, Zhiang Xie, Tao Du

**Affiliations:** The State Key Laboratory of Electronic Thin Films and Integrated Devices, School of Integrated Circuit Science and Engineering, University of Electronic Science and Technology of China, Chengdu 611731, China; chengyuh0106@foxmail.com (C.H.);

**Keywords:** in-memory computing, Hf_0.5_Zr_0.5_O_2_, ferroelectric memories, device-to-system perspective

## Abstract

Data movement has become a dominant bottleneck in modern artificial intelligence hardware, making in-memory computing a critical direction for energy-efficient and memory-centric architectures. Ferroelectric hafnium oxide provides a distinctive materials platform for this transition because field-driven polarization switching, non-volatility, CMOS compatibility, and nanoscale thickness scalability can be combined within a process-relevant oxide system. This review establishes a device-to-system perspective on HfO_2_-based and Hf_0.5_Zr_0.5_O_2_-based ferroelectric memories for in-memory computing. Instead of treating ferroelectric materials, memory devices, circuit primitives, and computing architectures as separate research topics, we examine how their mutual constraints define the achievable efficiency, precision, reliability, and scalability of hafnia-based computing systems. The discussion connects polarization engineering and defect control with charge-domain computation, threshold-state logic, associative search, analog weight representation, neuromorphic plasticity, and sensor-side processing. Particular emphasis is placed on the translation of ferroelectric functionality from individual devices to arrays, macros, and system-level accelerators. We identify variability, fatigue, charge trapping, multilevel-state uncertainty, peripheral overhead, and benchmarking inconsistency as the central barriers that prevent device-level advantages from directly becoming system-level gains. Finally, we outline a cross-layer roadmap in which ferroelectric stack engineering, variability-tolerant arrays, precision-scalable architectures, and SoC-level integration are co-optimized to enable reliable HZO-based memory-centric computing.

## 1. Introduction

In the burgeoning era of artificial intelligence (AI) and big data, the exponential growth in data volume has exposed the fundamental limitations of traditional von Neumann architectures. The physical separation of processing and memory units creates a “memory wall” bottleneck, where the energy and time costs of shuttling data between these units-often accounting for over 60% of total system power-far outweigh the energy consumed by computation itself. This inefficiency severely constrains the performance and scalability of modern computing systems. To overcome this challenge, in-memory computing (IMC) has emerged as a revolutionary paradigm, promising to break the von Neumann bottleneck by performing computations directly within the memory arrays, thereby minimizing or even eliminating costly data movement.

Among the various technologies enabling IMC, ferroelectric devices based on Hf_0.5_Zr_0.5_O_2_ (HZO) have garnered significant attention due to their immense potential to bridge the gap between novel device physics and industrial applications. Although SRAM-based and DRAM-based IMC benefit from mature CMOS integration and high endurance, their volatility, cell area, and standby or refresh overhead can limit energy efficiency in specific edge-computing scenarios. Although other emerging non-volatile memories like RRAM and MRAM show promise, they often face challenges regarding endurance, process variability, or complex integration. In contrast, ferroelectric memory technologies such as Ferroelectric Field-Effect Transistors (FeFETs), Ferroelectric Random-Access Memory (FeRAM), and Ferroelectric Tunnel Junctions (FTJs) offer non-volatility, high speed, and low operating voltage. Crucially, HZO-based ferroelectrics stand out because of their exceptional compatibility with standard Complementary Metal–Oxide–Semiconductor (CMOS) fabrication processes. Unlike conventional perovskite ferroelectrics, HZO can be deposited using atomic layer deposition (ALD) at temperatures compatible with back-end-of-line (BEOL) integration, enabling three-dimensional stacking and high-density integration without disrupting existing foundry flows. This CMOS compatibility, combined with scalability down to nanometer nodes, positions HZO as a superior candidate for next-generation IMC accelerators.

This review aims to provide a comprehensive overview of the path toward deployment of HZO-based IMC architectures, emphasizing the necessity of a co-design approach spanning from materials to systems. We begin by examining the fundamental material properties of HZO and the physical mechanisms underlying ferroelectric switching. Subsequently, we delve into various device implementations such as FeFETs and FTJs and analyze their respective performance metrics, including endurance, retention, and variability. The discussion then progresses to circuit-level innovations, showcasing different IMC array architectures and peripheral designs that leverage the unique characteristics of HZO devices. Finally, we explore system-level integration strategies and benchmark the performance of HZO-based accelerators against conventional systems. By synthesizing recent advancements across this entire spectrum, we also identify the critical bottlenecks and future research directions required to propel HZO-based IMC from academic research into commercial reality.

## 2. HfO_2_-Based Ferroelectric Materials

### 2.1. History of Research on HfO_2_-Based Ferroelectric Materials

The field of hafnia-based ferroelectrics was initiated by the 2011 report of switchable ferroelectric behavior in silicon-doped HfO_2_ thin films, which established fluorite-structured hafnia as a scalable and CMOS-compatible ferroelectric platform for non-volatile memories and emerging computing devices [1]. Compared with conventional perovskite-type ferroelectrics (such as PZT and SBT), HfO_2_-based ferroelectric materials exhibit remarkable advantages including high CMOS compatibility, excellent scalability, and lead-free environmental friendliness, thereby opening unprecedented avenues for non-volatile memory development [2,3].

After the emergence of ferroelectricity in Si:HfO_2_, the research focus rapidly expanded from phenomenological confirmation to the control of switching behavior, thermal stability, and device integration. Early electrical studies showed that polarization asymmetry in Si-doped HfO_2_ is strongly affected by interfacial effects, while the absence of a ferroelectric–paraelectric transition up to 478 K suggested a thermal stability window favorable for integrated devices [4]. The subsequent extension to differently doped HfO_2_ thin films established dopant engineering as an effective route to tune ferroelectric and antiferroelectric responses [2]. These material-level advances were soon translated into Si:HfO_2_-based FeFETs, linking fluorite-structured ferroelectricity with non-volatile transistor operation and laying the foundation for hafnia-based memory and in-memory computing technologies [5].

As research progressed, HZO gradually emerged as a mainstream composition because of its wider processing window and robust ferroelectric properties. The application potential of HZO-based materials for next-generation ferroelectric memories was further recognized in the context of CMOS back-end-of-line compatibility [3]. Meanwhile, intrinsic reliability issues in this material system, particularly “wake-up” and “fatigue” effects, became increasingly evident. In situ characterization during electric-field cycling revealed the structural evolution of HZO-based thin films, providing important experimental evidence for understanding the physical origins of these field-cycling effects [6]. In parallel, Si:HfO_2_ was identified as a fragile ferroelectric system, in which competing phase structures and strong sensitivity to annealing conditions highlighted the need for interface engineering and precise stoichiometric control [7].

With the maturation of hafnia-based ferroelectrics, the research focus shifted from establishing ferroelectricity toward performance optimization and reliability enhancement for memory applications. Critical assessments of HfO_2_-based ferroelectric thin films consolidated key reliability concerns, including wake-up, imprint, and fatigue endurance [8]. In parallel, rare-earth-element doping, particularly La incorporation, emerged as an effective approach to improve the reliability and FeRAM performance of HZO thin films [9]. For FeFETs, progress toward practical implementation at scaled technology nodes further highlighted parasitic charge trapping and reliability degradation as critical obstacles to device deployment [10]. At the array level, the development of hafnium oxide ferroelectric memories was increasingly framed as a transition from single-capacitor behavior to integrated memory arrays, emphasizing the need to connect materials optimization with scalable device operation [11].

The research scope of HfO_2_-based ferroelectric materials has further expanded beyond conventional non-volatile memories toward emerging applications such as neuromorphic computing, energy storage, and infrared sensing [12,13]. Comprehensive reviews of the field have framed this evolution around performance enhancement, materials design, and application-oriented device development [14]. Although intrinsic reliability issues, particularly wake-up and fatigue effects, remain unresolved, strategies such as ion irradiation, advanced doping, and interface engineering continue to improve cycling stability and operational lifetime [15,16].

### 2.2. Optimization of Ferroelectric Properties Through Interface Engineering

Interface engineering represents one of the most critical strategies for enhancing the performance of hafnia-based ferroelectric thin films. Given that these films are typically fabricated using atomic layer deposition (ALD), the quality of interfaces between the ferroelectric layer and the top/bottom electrodes directly influences the stability of the ferroelectric phase, polarization magnitude, and device reliability. In recent years, systematic investigations have been conducted from multiple perspectives, including interlayer insertion, electrode material optimization, and oxygen-vacancy regulation, yielding remarkable progress.

In interlayer engineering, oxide interlayers have been widely used to regulate the interface chemistry and ferroelectric response of HZO-based capacitors. The insertion of an ultrathin Al_2_O_3_ interlayer at the HZO/TiN interface can suppress oxygen-vacancy formation, yielding a remanent polarization of 42 μC/cm^2^ and endurance exceeding 10^8^ cycles [17]. Ozone-based interface pretreatment provides another effective route to reduce oxygen-vacancy-related defects and improve film crystallinity, enabling a remanent polarization as high as 73 μC/cm^2^ together with fatigue resistance over 10^8^ cycles [18]. These results indicate that interlayer-mediated defect passivation and phase control are effective strategies for enhancing the ferroelectric response and cycling stability of HZO thin films.

Electrode engineering provides another effective route for interface optimization in HZO-based ferroelectric capacitors. Although TiN electrodes offer good conductivity and process compatibility, their oxygen-scavenging behavior can promote oxygen-vacancy accumulation at the electrode/ferroelectric interface, thereby contributing to wake-up and fatigue degradation. Comparative studies of metal electrodes show that W electrodes can reduce interfacial oxygen-vacancy concentration, suppress wake-up behavior, and improve polarization endurance in HZO thin films [19]. Benefiting from the oxygen-regulating nature of W electrodes, W/HZO/W capacitors exhibit a high remanent polarization of 53.9 μC/cm^2^ together with improved cycling stability [20]. In addition, W incorporation into TiN electrodes can modulate the interface-state density, providing a further strategy to mitigate wake-up and fatigue effects [21].

**Figure 1:**
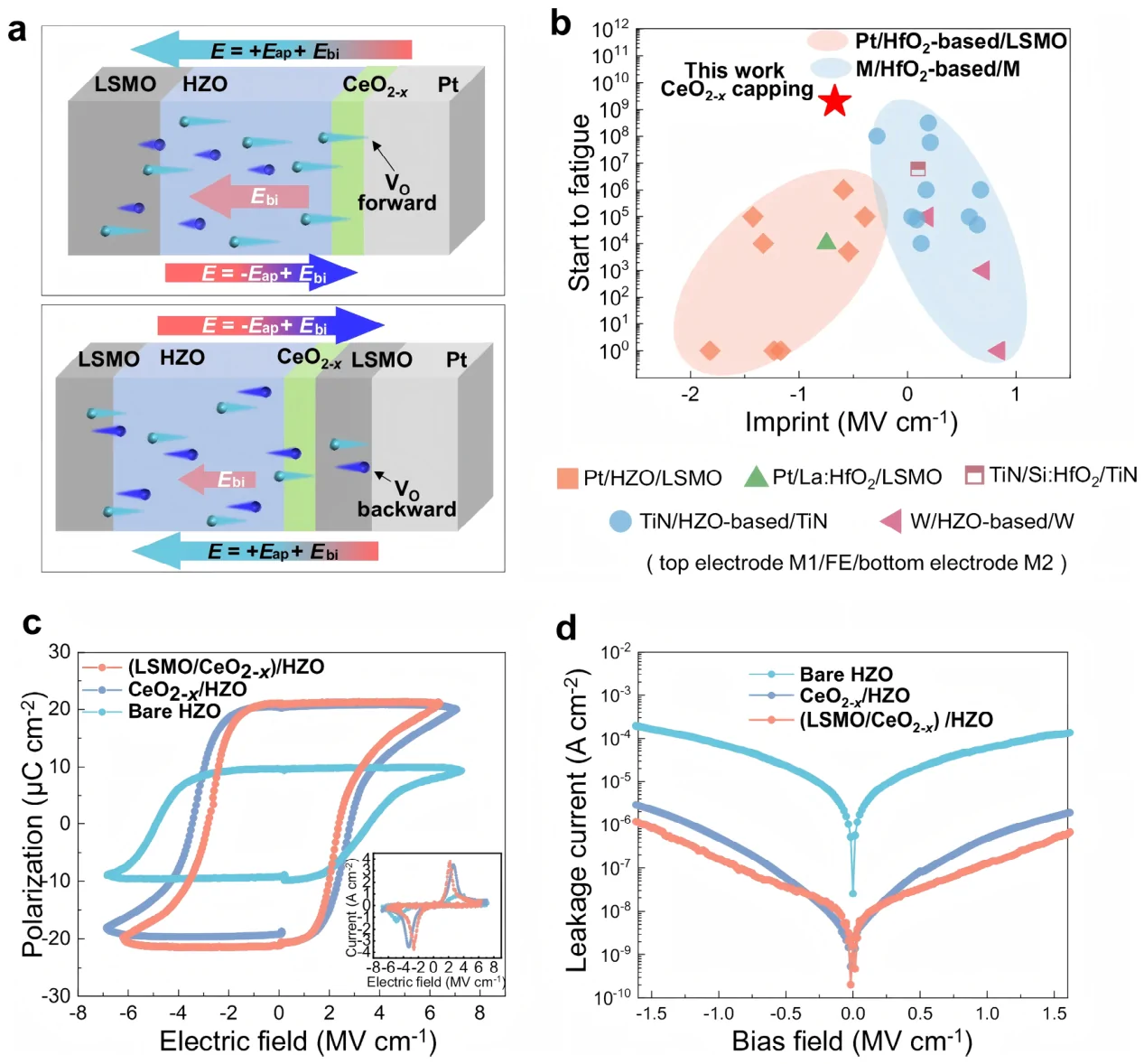
(**a**) Schematics depicting the impact of capacitor structure on the built-in bias (E_bi_) and its influence on the external electric fields applied in different orientations. The pink arrows within the schematic diagrams of the films denote the E_bi_, with their sizes indicating the magnitude of E_bi_. The multicolored arrows external to the films represent the composite electric field, formed by the superposition of E_bi_ and the applied electric field (E_ap_), with the length of the arrow proportionally representing the magnitude of the composite field. Spheres of varying colors are indicative of VO undergoing forward and reverse migration, respectively, with the length of the tails signifying the degree of mobility. (**b**) Illustration of fatigue behaviors for capacitors with various architectures and imprints. (**c**,**d**) Positive-up negative-down (PUND) curves and leakage current measurements for HZO devices with diverse structural designs. Reproduced from Ref. [22] under the terms of the Creative Commons Attribution-NonCommercial-NoDerivatives 4.0 International License (CC BY-NC-ND 4.0). © 2025 The Authors. Published by Springer Nature.

In terms of oxygen-vacancy regulation, interface engineering offers distinct advantages for suppressing fatigue degradation. A recent study represents a significant advance in this direction by constructing an oxygen-active CeO_2-X_/HZO heterointerface using pulsed laser deposition, in which the CeO_2-X_ layer functions as an oxygen-vacancy buffer and reservoir (Figure 1) [22]. During high-field cycling, oxygen vacancies are preferentially accommodated in the CeO_2-X_ layer rather than migrating within the HZO ferroelectric layer, thereby preventing the formation of conductive filaments and defect clusters and effectively suppressing polarization fatigue. The symmetric electrode design further enables synergistic optimization of the top and bottom interfaces, resulting in fatigue-free ferroelectric switching exceeding 10^11^ cycles and an exceptional endurance lifetime beyond 10^12^ cycles [22]. This study elucidates the physical mechanism of fatigue suppression through interface engineering at the atomic scale: high-resolution transmission electron microscopy and electron energy loss spectroscopy directly reveal the interfacial oxygen-vacancy distribution, while first-principles calculations clarify how the CeO_2-X_/HZO heterointerface modulates oxygen-vacancy formation energy. This work not only provides new insight into fatigue mechanisms in hafnia-based ferroelectrics but also offers a generalizable interface-engineering strategy for designing high-reliability ferroelectric devices.

### 2.3. Optimization of Ferroelectric Properties in Hafnium Oxide via Doping

Elemental doping is a central strategy for stabilizing the ferroelectric phase and tailoring the electrical properties of HfO_2_-based thin films. By introducing dopants with different valence states and ionic radii, key factors such as oxygen-vacancy concentration, lattice stress, and phase-boundary stability can be effectively regulated, thereby modulating remanent polarization, coercive field, and endurance behavior. Among various dopant families, rare-earth elements, particularly lanthanide dopants, have attracted considerable attention because of their pronounced ability to stabilize the ferroelectric phase and improve the overall functional properties of hafnia-based ferroelectrics.

Among rare-earth dopants, La doping has become one of the most extensively investigated strategies for improving the ferroelectricity and reliability of HfO_2_-based thin films. In ultrathin HZO films, precise control of the La concentration within the range of 4–6% enables a high remanent polarization of 2P_r_ > 30 μC/cm^2^, low-voltage operation below 1 V, and ultrahigh endurance exceeding 3 × 10^11^ cycles in 5 nm films [23]. The incorporation of La^3+^ regulates oxygen-vacancy formation, suppresses monoclinic-phase formation, and promotes the preferred (111) orientation, thereby lowering the coercive field while maintaining good thermal stability of the ferroelectric response. These effects provide important materials-design guidelines for developing low-power and high-reliability ferroelectric memory devices. In epitaxial HfO_2_ films, optimization of the La concentration further confirms the effectiveness of this doping strategy, with 5% La doping yielding a remanent polarization above 20 μC/cm^2^, a reduced coercive field, and endurance exceeding 10^10^ cycles [24]. Moderate La doping at approximately 0.7% can also mitigate the wake-up effect, enabling field-cycling endurance up to 10^11^ cycles while maintaining a high remanent polarization of at least 25 μC/cm^2^ [25].

Beyond La doping, other rare-earth dopants also provide distinct routes for phase stabilization and property optimization. For thick-film applications of approximately 100 nm, Y doping can effectively stabilize the ferroelectric phase of HfO_2_ by promoting columnar grain microstructures, thereby offering superior thickness scalability compared with La doping [26]. First-principles comparisons of group-III dopants, including La, Y, Al, and Gd, further indicate that La provides the most robust ferroelectric phase stabilization in HZO. This effect is associated with an increased oxygen-vacancy formation energy, which contributes to improved endurance and reduced susceptibility to dielectric breakdown [27].

Al and Si doping, as established dopant-engineering strategies, also play important roles in optimizing the ferroelectric properties of HfO_2_-based thin films. By tailoring the Al distribution within HZO films, remanent polarization, leakage current, and breakdown voltage can be significantly improved [28]. The optimized Al-doped films exhibit robust endurance associated with reversible phase transitions, highlighting their potential as reliable materials for memory applications. In 10 nm HfO_2_ films, combined Al and Si doping further yields a remanent polarization of approximately 20 μC/cm^2^ with endurance exceeding 10^8^ cycles [29]. More broadly, dopants such as Zr^4+^, La^3+^, Y^3+^, and Si^4+^ provide effective routes to enhance polarization, phase stability, and electrical performance, offering important guidance for CMOS-compatible ferroelectric implementations [30].

## 3. HfO_2_-Based Ferroelectric Devices and In-Memory Computing Circuits

### 3.1. Hafnia-Based Ferroelectric Capacitors (FeCAPs)

#### 3.1.1. FeCAP/FeRAM Cell Operation and Capacitive Readout

Hafnia-based ferroelectric capacitors (FeCAPs) are two-terminal metal–ferroelectric–metal (MFM) devices in which binary information is encoded by the two stable remanent-polarization orientations. A voltage pulse exceeding the coercive voltage sets the polarization state. In a conventional 1T-1C FeRAM cell, an access transistor connects the selected FeCAP to the bit line, and the stored state is detected from the polarization-dependent charge generated during a read pulse. Because conventional charge-based sensing can reverse one of the polarization states, the detected data must be restored after readout. BEOL-integrated 16 kb HfO_2_:Si 1T-1C arrays have demonstrated that this cell architecture can provide scalable, high-speed non-volatile memory operation [31,32].

#### 3.1.2. Asymmetric FeCAP Arrays for IMC

Asymmetric FeCAPs enable non-destructive readout through polarization-dependent small-signal capacitance. Dissimilar electrode interfaces yield distinguishable capacitances for the two polarization states at low or zero DC bias. A subcoercive signal senses the resulting displacement–charge difference without polarization reversal, thereby reducing read disturbance [33,34].

In capacitive IMC arrays, non-volatile capacitance states encode weights and input-voltage pulses represent vector elements. Here, capacitive coupling denotes displacement–charge transfer: a transition on the plate line (PL) induces charge on the bit line (BL), where contributions from selected cells accumulate to perform parallel multiply–accumulate (MAC) and vector–matrix multiplication (VMM) operations [34,35,36]. Multi-state capacitance supports analog or multibit weights [37].

Array computation proceeds in two phases: column integration capacitors are initialized to a common-mode voltage, after which row-voltage steps generate displacement charges that accumulate along each column. The resulting voltage is selected by a multiplexer, digitized by an ADC, and stored [34,35,36]. Negligible DC read current reduces static power, sneak paths, and IR drop, enabling selector-free operation; however, the modest capacitance on/off ratio and kT/C-limited noise increase sensing and data-conversion demands [34,35].

**Figure 2:**
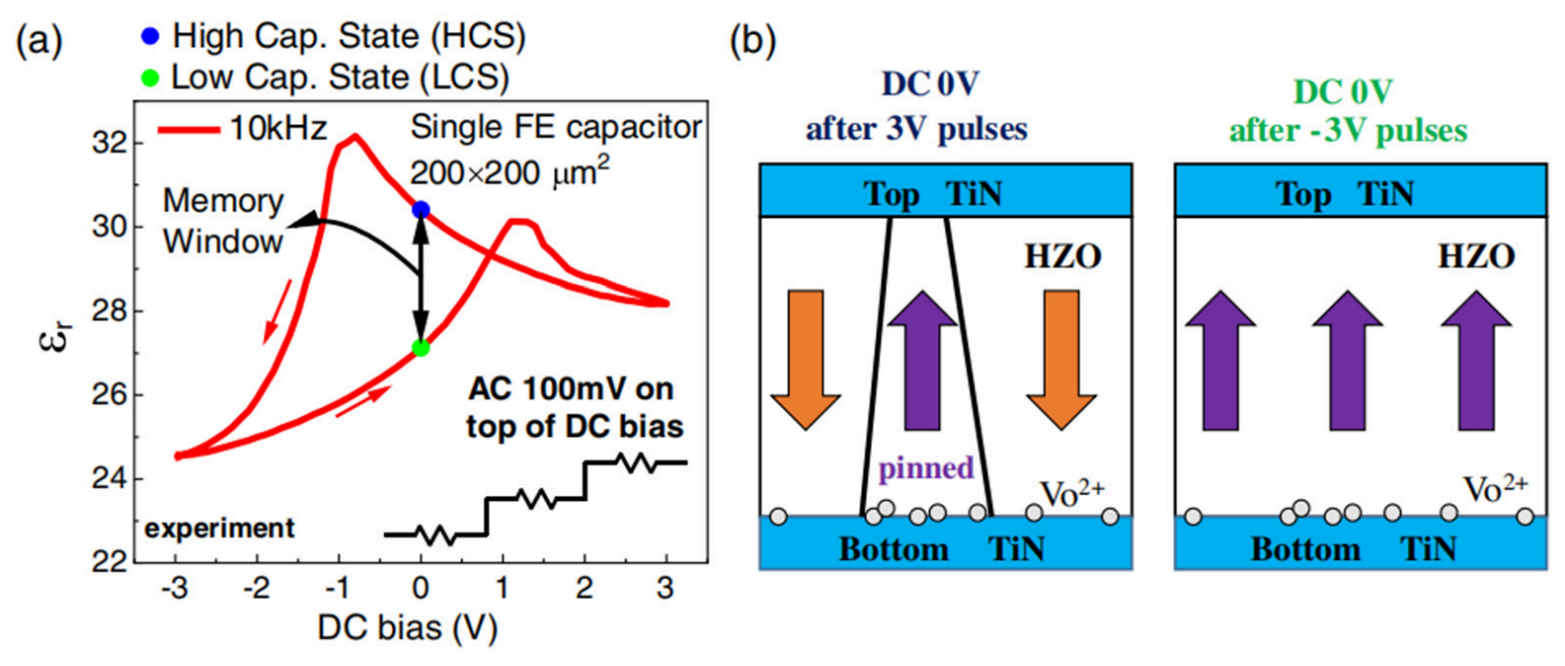
(**a**) Asymmetric small-signal C–V shows a memory window at DC 0 V with 10 kHz 100 mV AC small signals applied. (**b**) Physical illustration of the asymmetric C–V at DC 0 V. The positively charged oxygen vacancies (Vo^2+^) at the BE pinned some domains up-polarized. While +3 V pulses tend to flip the domains down-polarized, more DWs are formed due to the pinned domains, resulting in higher small-signal capacitance. The pinned up-polarized domains do not create DWs as −3 V pulses tend to flip the domains up-polarized too, resulting in lower capacitance. Reproduced from Ref. [34] under the terms of the Creative Commons Attribution 4.0 International License (CC BY 4.0). © 2022 The Authors. Published by Wiley-VCH GmbH.

Figure 2 illustrates the zero-bias capacitance window produced by oxygen-vacancy-induced domain-wall pinning at asymmetric electrode interfaces, which yields an on/off ratio exceeding 110% at 0 V [34]. Positively charged vacancies near the bottom electrode pin upward-polarized domains; switching against this direction creates more domain walls and a larger capacitance, whereas switching along it produces fewer domain walls and a lower capacitance. This behavior enables non-destructive weight sensing during inference.

The 1T-1C unit cells serve as fundamental building blocks for large-scale FeRAM arrays integrated with CMOS logic [31,38]. Recent 16 kb 1T-1C FeRAM arrays implemented in 130 nm BEOL technology demonstrate competitive programming speeds down to 4 ns, endurance exceeding 10^7^ cycles, and solder-reflow compatibility required for industrial packaging [31]. Circuit schematics of asymmetric capacitor arrays further illustrate the distinct voltage-biasing schemes applied to WLs, BLs, and PLs to optimize charge-transfer efficiency [10]. Die micrographs reveal dense integration with critical dimensions compatible with advanced technology nodes, while Convolutional Neural Network (CNN) inference demonstrations based on charge-domain accumulation show energy-efficiency advantages over SRAM-based architectures [34].

3D stacking and BEOL integration provide critical pathways for increasing the storage density of capacitive IMC systems [39]. The thermal stability of HZO-based FeCAPs enables processing temperatures compatible with BEOL integration below 400 °C, thereby facilitating monolithic 3D integration with logic layers. Solder-reflow testing at 260 °C further confirms the robustness of these capacitors against the thermal stress encountered during standard packaging processes [31]. Interfacial asymmetry enables a non-destructive capacitive memory window of approximately 4.7× at zero DC bias, while optimized nonzero read bias can further increase the window to approximately 8× [33,40].

### 3.2. Hafnia-Based FeFETs for IMC

#### 3.2.1. Structure and Storage Mechanism Optimization of FeFET

Gate-stack electrostatics and the resulting memory window directly influence the operating margin and reliability of HfO_2_-based FeFETs under device variation and temperature changes [39,41,42]. Within an FeFET gate stack, voltage coupling denotes the electrostatic transfer of the applied gate bias to the semiconductor surface potential; its strength is determined by voltage division among the ferroelectric layer, dielectric interlayers, and semiconductor capacitance. A gate-side Al_2_O_3_ interlayer can enlarge the memory window by introducing trapped charge at the Al_2_O_3_/HZO interface with a polarity opposite to that at the HZO/SiO_x_ interface, although interlayer thickness and charge trapping can affect retention and endurance [43]. Interfacial-layer engineering can reduce interface-state density and modify the balance between memory-window enlargement and charge-trapping-induced degradation [43].

Read-disturb immunity is an important requirement for dense FeFET arrays. Drain-erase operation and independently controlled read gates can reduce read-induced threshold-voltage disturbance by separating polarization programming from the read path [33,44,45]. Dual-port architectures with separate write and read gates further enable disturb-free operation and amplified memory windows, although their trade-offs in search-energy efficiency and array-level implementation remain under investigation [44,45,46,47,48].

#### 3.2.2. FeFET-Based Logic-in-Memory Architectures

Logic-in-memory architectures based on FeFETs enable computation and storage to be colocated within a single transistor cell, thereby reducing data movement associated with the von Neumann bottleneck. The intrinsic hysteresis of FeFETs allows Boolean logic functions to be implemented through single-device configurations by programming the polarization state and modulating terminal voltages [49,50]. Compact circuit implementations have further demonstrated 2T FeFET XOR units and non-volatile majority gates for full-adder operation, achieving substantially reduced device counts compared with conventional CMOS counterparts [51,52]. Reconfigurable logic circuits that exploit interacting polarization-switching and charge-trapping effects further extend FeFET functionality, enabling multiple Boolean operations, including 16 logic functions, within compact cell footprints [53].

Building upon these compact logic primitives, recent research has further advanced the field by proposing a comprehensive analog-to-digital converters (ADC)-free FeFET Computing-in-Memory architecture tailored for energy-efficient and low-latency edge AI computing [54]. Unlike conventional designs that suffer from the area and power overhead of ADC, this design leverages the non-volatile logic capability of FeFETs to execute computations directly.

**Figure 3:**
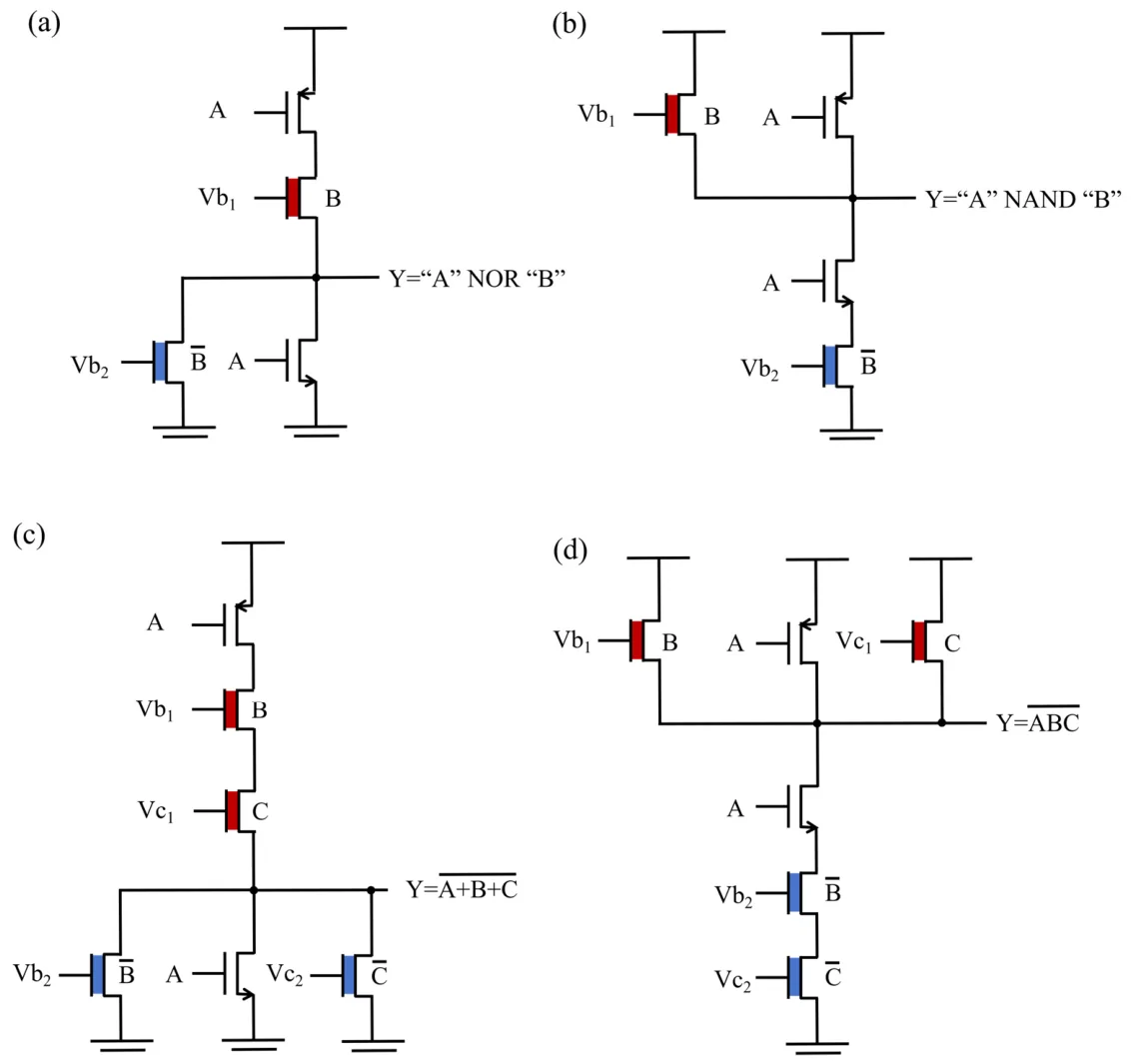
(**a**) Structure diagram of the FeFETs-CiM NOR CMOS circuit. (**b**) Structure diagram of the FeFETs-CiM NAND CMOS circuit. (**c**) Structure diagram of the FeFETs-CiM 3-bit NOR CMOS circuit. (**d**) Structure diagram of the FeFETs-CiM 3-bit NAND CMOS circuit. The red FeFET gates denote devices programmed to store the weight bits B and C, whereas the blue FeFET gates denote devices programmed to store their logical complements, respectively. Adapted from Ref. [54] under the terms of the Creative Commons Attribution 4.0 International License (CC BY 4.0). © 2026 The Authors.

The fundamental building blocks of this architecture are illustrated in Figure 3, which presents the schematic diagrams of FeFET-CMOS hybrid logic gates. By integrating FeFETs with conventional CMOS transistors, the architecture implements basic Boolean functions, including 2-bit NOR, 2-bit NAND, 3-bit NOR, and 3-bit NAND gates. In these configurations, the FeFETs act as non-volatile input or control elements, exploiting their hysteretic I–V characteristics to store weights and participate in logic evaluation within the same compact cell.

**Figure 4:**
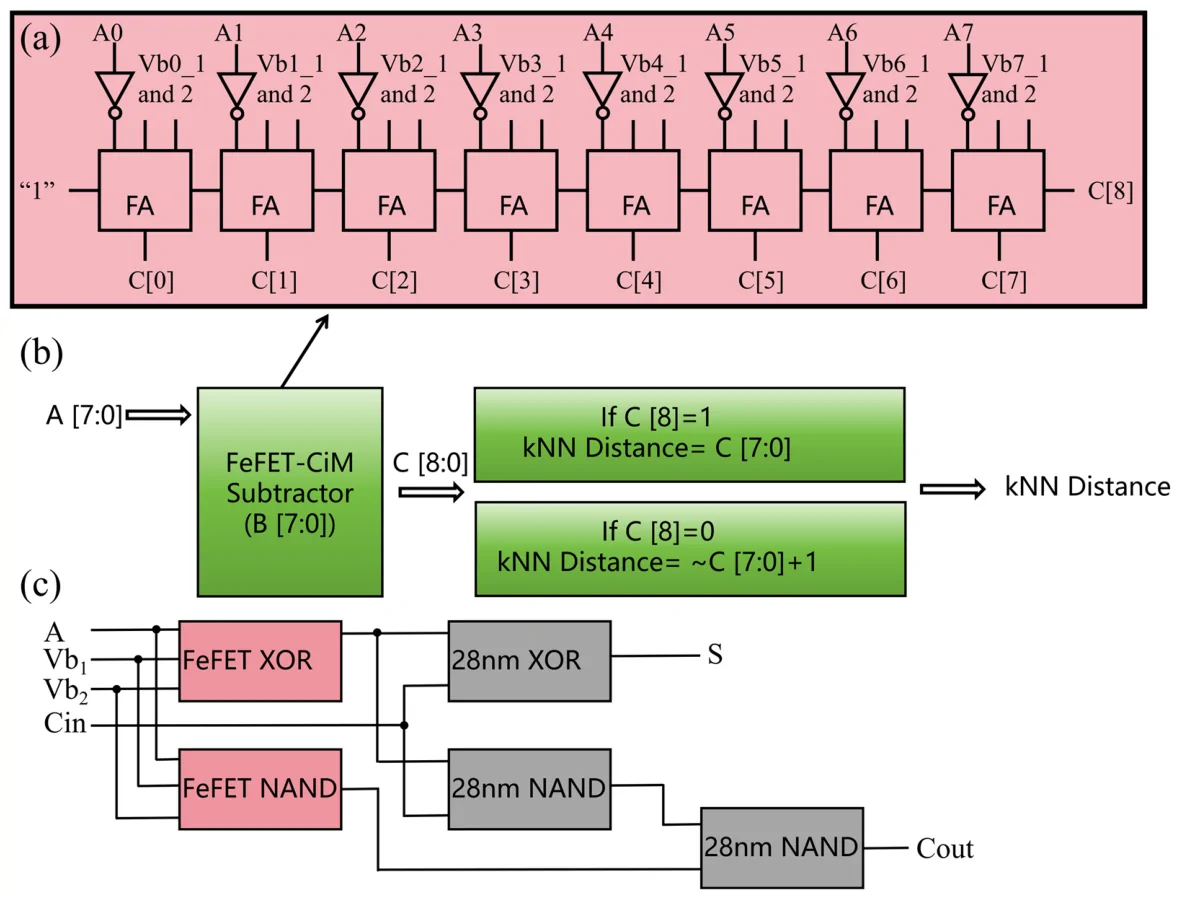
(**a**) Structure diagram of the application-specific FeFETs-CiM kNN 8-bit subtractor; (**b**) 8-bit kNN distance calculator based on application-specific FeFETs-CiM kNN 8-bit subtractor; (**c**) structure diagram of the FeFETs-CiM full-adder circuit. Adapted from Ref. [54] under the terms of the Creative Commons Attribution 4.0 International License (CC BY 4.0). © 2026 The Authors.

Building on these versatile logic gates, the architecture further demonstrates a practical bit-serial FeFET-CiM subtractor, as shown in Figure 4. Figure 4a presents an 8-bit subtractor constructed by cascading full-adder units, where the logic operations are mapped onto the FeFET array to compute the difference between input vectors. This implementation is tailored for the k-Nearest Neighbors (kNN) distance calculation flow in Figure 4b, in which the subtractor directly computes the Manhattan distance in the digital domain. The circuit-level implementation of a single full adder, composed of the proposed FeFET XOR and NAND gates together with CMOS logic, is shown in Figure 4c. This design validates the feasibility of FeFET-based IMC architectures for accelerating edge-intelligence workloads with high precision and low latency.

#### 3.2.3. FeFET-Based Ternary Content-Addressable Memory (TCAM)

Ternary content-addressable memory (TCAM) represents an important application domain for FeFETs by enabling high-speed pattern matching against stored entries. FeFET-based TCAM designs exploit non-volatile threshold-voltage modulation to encode ternary states (0, 1, X) within compact cell configurations, such as 2FeFET–2T and 2FeFET-1T architectures [35,40,51,55]. Charge-domain TCAM implementations further sense the match line through capacitive charge transfer, reducing search energy relative to current-domain alternatives, while cross-coupled match-line structures improve search yield and tolerance to process variation [56,57].

Temperature resilience and device-variation tolerance are essential for automotive and Internet of Things (IoT) deployments. Experimental characterization across process corners and temperatures from 25 °C to 120 °C shows stable search-delay distributions and energy efficiency, with binary CAM cells maintaining robust retention and search accuracy despite MW degradation at elevated temperatures [41,42,58,59]. To address the persistent challenges of write disturb and process-variation sensitivity in ferroelectric TCAM, circuit-level innovations have been introduced to enhance reliability [58]. The cross-coupled 4T2F architecture strategically employs transmission gates as access devices, enabling full voltage transfer during programming to eliminate write failures while isolating the gate-source voltage of unselected cells to suppress write disturb. In addition, a dedicated match-line (ML) scheme improves energy efficiency by dynamically controlling the discharge path and reducing the effective ML capacitance. This design mitigates charge-sharing effects in conventional TCAM structures and provides a robust sensing voltage margin. Such architectural refinements underscore the potential of FeFET-based TCAMs to achieve high search yields under stringent process variations.

Statistical analysis of ML voltage distributions provides further insight into the noise immunity of different FeFET TCAM architectures. Compared with previous designs, the proposed 4T2F architecture exhibits a nearly ideal voltage distribution: the match-state voltage is tightly clustered near VDD with minimal variation, whereas the mismatch state is fully discharged. This improved separation originates from the cross-coupled structure, which eliminates floating nodes, and the transmission-gate access mechanism, which enables precise voltage control during search operation. The clear distinction between match and mismatch states validates the robustness of the architecture against circuit noise and manufacturing-induced variations.

### 3.3. Hafnia-Based Ferroelectric Metal Field-Effect Transistors (FeMFETs) for IMC

#### 3.3.1. Structure and Operating Principle of FeMFET

The ferroelectric metal field-effect transistor (FeMFET) represents a distinct architectural evolution from the conventional FeFET through the insertion of an intermediate metal layer between the ferroelectric film and the underlying gate dielectric. This metal–ferroelectric–metal–insulator–semiconductor (MFMIS) configuration physically decouples ferroelectric polarization switching from the semiconductor channel, thereby providing greater flexibility in gate-stack design and improved device reliability. Unlike conventional FeFETs, in which the ferroelectric layer is integrated with the gate dielectric and electrostatically modulates the channel, the FeMFET employs an internal metal gate as a floating electrode, forming a capacitive voltage divider between the MFM capacitor and the underlying MOS capacitor.

The operation of HfO_2_-based FeMFETs is governed by this capacitive voltage-division mechanism, through which the polarization state of the MFM capacitor modulates the channel surface potential. The physical separation between the ferroelectric layer and the semiconductor channel also enables non-destructive readout because the stored polarization state can be sensed through the drain current at gate voltages well below the coercive switching field [60]. This read mechanism avoids polarization reversal during sensing and therefore differs fundamentally from the destructive readout typically associated with conventional FeRAM.

Electrical demonstrations further confirm the effectiveness of the separated-capacitor architecture. FeMFETs with an optimized capacitance ratio have achieved a memory window of approximately 0.7 V under program/erase voltages of 6 V, together with stable operation over 10^6^ field cycles under symmetric pulse conditions [60,61]. At the array level, an 8 kb FeMFET test chip has demonstrated programming speeds down to 10 ns and clearly separated threshold-voltage states, indicating the potential of this architecture for scalable and high-speed non-volatile memory implementation [60,61,62].

#### 3.3.2. Advantages and Design Trade-Offs Relative to FeFETs

The separated-capacitor architecture of FeMFETs offers several critical advantages over conventional FeFETs, particularly in endurance, retention, and operational reliability. The primary benefit arises from eliminating direct electrical stress on the gate insulator during polarization switching. In conventional FeFETs, the high electric fields required for ferroelectric switching also stress the thin interfacial oxide, leading to charge trapping, interface-trap generation, and eventual device degradation [63]. In contrast, the FeMFET architecture confines the high switching field mainly within the MFM capacitor, thereby mitigating degradation mechanisms in the underlying MOS transistor [64].

Endurance is a particularly important advantage of the separated-capacitor FeMFET architecture. Vertical-pillar FeMFET implementations have demonstrated memory windows exceeding 3 V and program/erase speeds of approximately 100 ns, while offering substantially improved endurance compared with planar FeFET counterparts [64]. By optimizing the capacitance ratio between the MFM and metal–insulator–semiconductor (MIS) components, the electric field across the gate insulator during switching can be reduced, directly improving device longevity. In addition, the separated configuration facilitates interface-engineering strategies, including nitrogen radical treatment and thermal annealing, which improve retention by mitigating charge accumulation at the ferroelectric–insulator interface [63].

The decoupling of polarization and charge-trapping dynamics has been investigated using in situ midpoint-voltage extraction. By correlating the dynamic response with the DC component of the drain current, FeMFETs enable independent characterization of ferroelectric polarization switching and charge-trapping processes during device operation [63]. This capability provides important insight into degradation mechanisms that are otherwise convoluted in conventional FeFETs and supports more precise device optimization and reliability assessment.

However, the FeMFET architecture also introduces design complexities associated with the floating intermediate node. The additional metal electrode can increase the depolarization field in the ferroelectric layer, making careful capacitance-ratio optimization essential for achieving favorable subthreshold-swing behavior [61]. In particular, the trade-off between increasing the voltage drop across the MFM capacitor and maintaining sufficient channel inversion strength requires sophisticated gate-stack engineering, as explored in various design-technology co-optimization (DTCO) methodologies [65].

#### 3.3.3. FeMFET-Based IMC Circuits

FeMFET-based IMC architectures have attracted increasing interest by combining non-volatile storage with improved reliability enabled by the separated-capacitor structure. The 1T-1C configuration, in which an MFM ferroelectric capacitor is integrated in the back-end-of-line (BEOL) interconnect layers and electrically connected to the gate of a logic transistor, represents a promising route toward high-density memory arrays [62]. This architecture supports functional memory-array operation and accurate MAC computation, demonstrating the feasibility of FeMFET-based IMC for neural-network inference applications [62,66].

The structural versatility of FeMFETs has also motivated their exploration in multilevel-cell (MLC) 3D NAND flash applications. FeMFET designs optimized for MLC operation can provide high-density storage while improving endurance relative to conventional FeFET-based approaches [66]. The separated-capacitor configuration enables more precise control of threshold-voltage distributions, thereby supporting reliable multibit storage per cell. DTCO methodologies have further been applied to enhance 1T-1C FeMFET bitcells, allowing systematic exploration of the design space and identification of optimized configurations for specific application requirements [65]. This approach supports the customization of FeMFET arrays for diverse IMC workloads, ranging from high-precision neural-network inference to energy-constrained edge computing. Moreover, unlike conventional FeRAM, which relies on destructive readout, the intrinsic non-destructive read capability of FeMFETs is naturally compatible with CiM architectures, where frequent and iterative read operations are essential for computational efficiency.

### 3.4. Hafnia-Based FTJs for IMC

**Figure 5:**
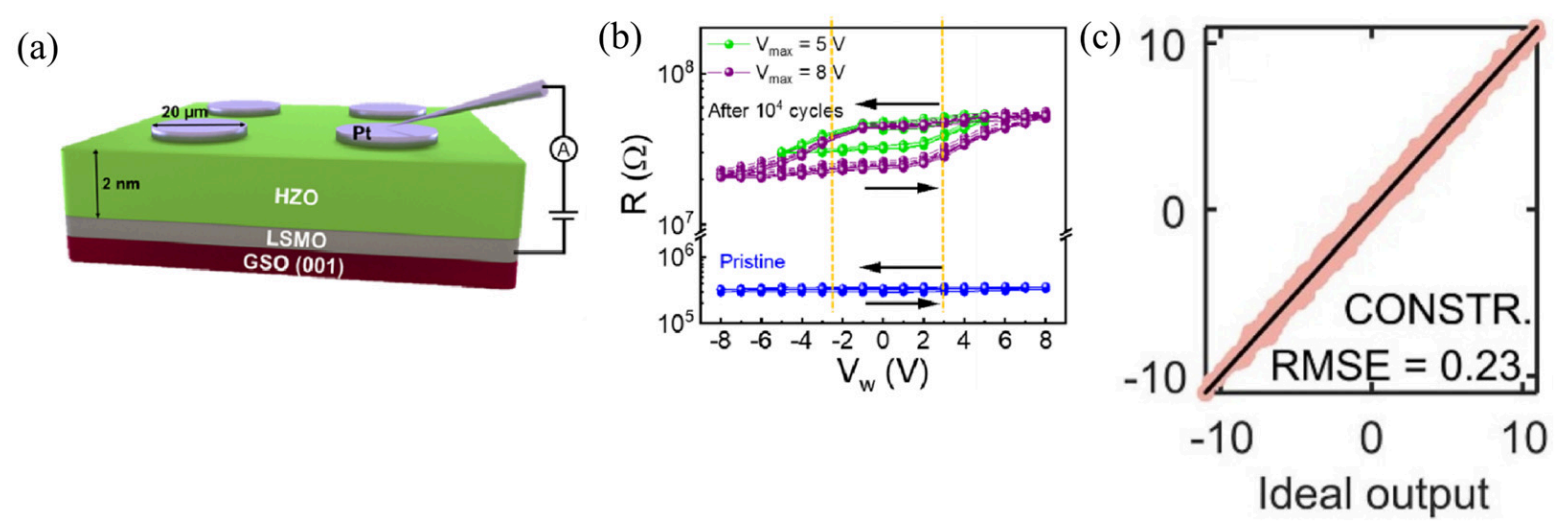
(**a**) Sketch of the 2 nm HZO tunnel-junction stack. (**b**) Dependence of junction resistance on the writing voltage measured in the pristine junction and after 10^4^ cycles. Adapted from Ref. [67]. Copyright © 2021 American Chemical Society. Licensed under CC BY 4.0. (**c**) Matrix–vector multiplication in the CONSTRICTED operating mode. Adapted from Ref. [71]. Copyright © 2025 The Author(s). Published by IOP Publishing Ltd. Licensed under CC BY 4.0.

HZO FTJs are attractive two-terminal devices for IMC and neuromorphic hardware because they combine non-volatile polarization switching with resistive and non-destructive readout. As illustrated in Figure 5a, a typical HZO FTJ consists of an ultrathin ferroelectric HZO layer sandwiched between two electrodes. Reversal of the ferroelectric polarization modifies the effective tunneling barrier and thereby changes the junction conductance. This polarization-controlled resistance modulation is shown in Figure 5b, where the resistance state is programmed by the applied write voltage. In epitaxial 2 nm HZO tunnel junctions, ferroelectric switching and electroresistance have been shown to be closely correlated, while pulse-driven conductance modulation enables memristive behavior, spike-timing-dependent plasticity (STDP), and retention [67]. The low-current nature of HZO FTJs has further been exploited for deep-learning acceleration, where sub-nA operation provides an energy-efficient route toward neural-network hardware [68].

A central requirement for practical HZO FTJ computing arrays is the transition from individual junction operation to reproducible device populations. CMOS-compatible HZO FTJs fabricated on a 300 mm wafer platform have demonstrated nanoscale 100 × 100 nm^2^ cells, multilevel storage, endurance up to 10^6^ cycles, and switching yields above 80%, indicating their compatibility with industrially relevant process flows [69]. In parallel, the analog programmability of FTJ conductance has been exploited for IMC applications. W/HfZrO_4_/TiN FTJ memristors with 60 programmable conductance states have been evaluated as resistive processing units in crossbar-based IMC accelerators [70]. System-level simulations based on experimentally calibrated W/HfZrO_4_/TiN FTJs further clarify how different operating modes influence analog matrix–vector multiplication. In the CONSTRICTED mode shown in Figure 5c, the computed output closely follows the ideal MVM result; however, this improvement is achieved by restricting the usable dynamic range, thereby requiring careful control of programming noise, read noise, and peripheral precision [71].

HZO FTJs have also been extended from vector–matrix multiplication to associative search. Experimentally demonstrated HZO FTJ arrays exhibit sufficiently distinguishable low-resistance-state (LRS) and high-resistance-state (HRS) current distributions to support array-level search operations. Based on such arrays, combination-encoding content-addressable memory (CECAM) has been implemented using HZO FTJs [72]. By encoding each word as a fixed-number combination of LRS and HRS devices and identifying the matching entry through the minimum ML current, this approach increases the content density to 0.75 bit per switch, exceeding the 0.5 bit per switch limit of conventional two-resistor CAM cells. This capability makes HZO FTJ arrays particularly relevant for AI-CAM, similarity-search, and event-routing workloads, although the nA-level match current imposes stringent requirements on sensing latency and peripheral-circuit design.

For neuromorphic computing, HZO FTJs naturally function as artificial synapses because partial polarization switching enables gradual conductance modulation. Epitaxial HZO FTJ synapses have achieved an ON/OFF ratio above 500, a conductance window tunable over 1–250 ns, long-term potentiation/depression (LTP/LTD), STDP, and 93.7% MNIST recognition accuracy [73]. Interface-engineered ITO/HZO/WO_X_ FTJs further improve endurance beyond 10^8^ cycles and provide 64 distinguishable conductance states, supporting stable spike-dependent plasticity and MNIST inference [74]. Self-rectifying FTJ design represents another important direction, where HfO_2_/ZrO_2_/HfO_2_ superlattice FTJs combine a large ON/OFF ratio with strong rectification to suppress sneak-path currents in passive crossbar arrays [75]. Experimentally measured conductance-update characteristics can also be incorporated into neural-network simulations to evaluate the impact of device non-idealities on recognition performance. Beyond conventional excitatory synapses, dual-modal HZO FTJs have demonstrated excitation/inhibition switching, BCM-like learning, pain-perception emulation, and image edge recognition, suggesting a route toward more bio-inspired sensing and perceptual-computing functions [76]. Multi-state TaN/(Hf,Zr)O_2_/Ta FTJs with more than 32 resistance states further support the feasibility of HZO FTJs as weighted elements for IMC arrays and vector–matrix multiplication [77].

Several challenges still hinder the transition of HZO FTJs from promising device demonstrations to large-scale computing macros. First, device design must balance ferroelectric stability, read current, and tunneling electroresistance (TER) ratio: thinner HZO barriers increase the tunneling current but also enhance sensitivity to leakage, interface quality, and phase instability, whereas thicker barriers improve polarization robustness at the expense of reduced read current. Second, analog computing requires conductance updates that are linear, symmetric, and reproducible; however, FTJ analog states originate from partial domain switching and parallel conduction paths, which inherently constrain the usable dynamic range. Third, array-level operation introduces device-to-device variability, read disturbance, sneak currents, low ML currents, and peripheral-circuit overhead. Finally, most HZO FTJ-based IMC demonstrations remain confined to single devices, small arrays, or simulation-assisted evaluations. Future progress will therefore require co-optimization of ferroelectric stack engineering, electrode and interface design, selector-free array operation, peripheral sensing circuits, and hardware-aware algorithms.

### 3.5. Cross-Device Comparison of Hafnia-Based Ferroelectric Memory Platforms

**Figure 6:**
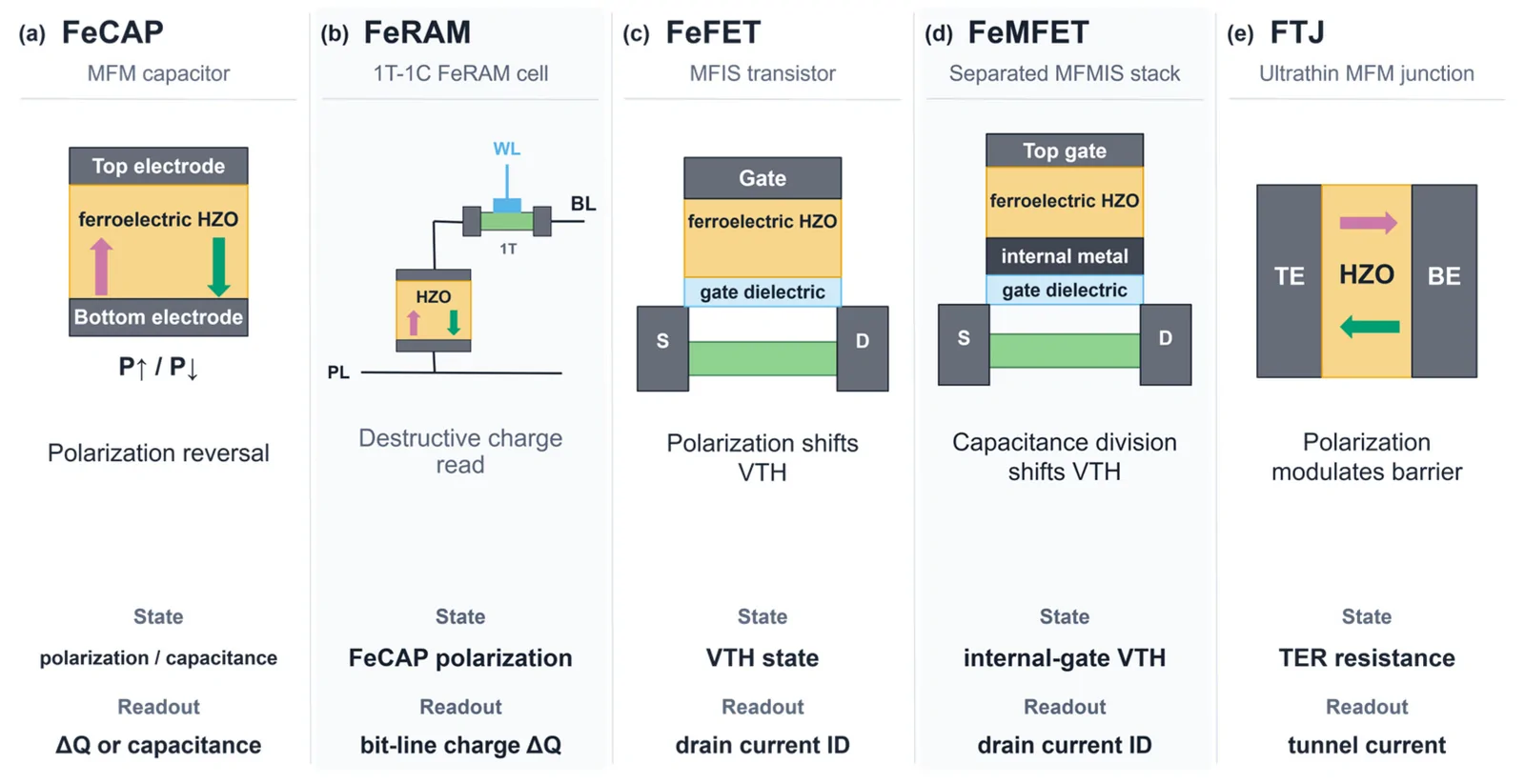
Comparative topologies and state–readout pathways of hafnia-based ferroelectric memory devices. (**a**) FeCAP with a metal–ferroelectric–metal (MFM) stack. (**b**) A 1T–1C FeRAM cell comprising an access transistor and a FeCAP. (**c**) FeFET with a metal–ferroelectric–insulator–semiconductor (MFIS) gate stack. (**d**) FeMFET with a separated-capacitor or metal–ferroelectric–metal–insulator–semiconductor (MFMIS) configuration. (**e**) FTJ with an ultrathin ferroelectric tunneling barrier. The stored state and corresponding electrical readout variable are indicated for each device.

Figure 6 places the five principal hafnia-based ferroelectric memory platforms on a common device-level basis. A FeCAP is a two-terminal metal–ferroelectric–metal (MFM) element in which polarization is written by an electric field and the stored state is represented by remanent polarization or a polarization-dependent capacitance. A FeRAM cell adds an access transistor to the FeCAP, most commonly in a 1T–1C topology, to select the storage capacitor during write and read operations; FeRAM therefore denotes the selected memory-cell organization rather than a distinct ferroelectric stack [31,32,33,34]. A FeFET integrates the ferroelectric layer into a metal–ferroelectric–insulator–semiconductor (MFIS) gate stack, so that polarization changes the channel threshold voltage [39,43]. By contrast, a FeMFET inserts an internal metal electrode and separates the MFM ferroelectric capacitor from the underlying MOS gate stack. The resulting MFMIS or separated-capacitor configuration transfers the polarization state to the channel through electrostatic voltage division [60,61]. An FTJ returns to a two-terminal topology but uses an ultrathin ferroelectric layer as the tunnel barrier; polarization reversal modifies the barrier profile and produces tunneling electroresistance (TER) [67].

These topological differences determine how the stored state is read. In a conventional symmetric FeCAP or 1T–1C FeRAM cell, a read pulse generates a polarization-dependent switching charge; because one logic state may be reversed during sensing, the read is destructive and must be followed by data restoration [31,32]. An asymmetric FeCAP instead distinguishes the two states through their small-signal capacitance or displacement–charge response at a subcoercive bias, enabling non-destructive charge-domain readout [33,34]. FeFETs and FeMFETs are also read non-destructively: a gate voltage below the coercive switching condition converts the stored polarization state into a threshold-voltage-dependent drain current [44,45,60,61]. For an FTJ, a low read bias probes the polarization-dependent tunnel current or resistance without intentionally switching the ferroelectric barrier [67,68,69,70,71].

The major advantages and limitations follow directly from these transduction mechanisms. FeCAPs combine a compact two-terminal structure, negligible DC read current, natural column-wise charge accumulation, and compatibility with back-end-of-line integration, making them attractive for low-static-power capacitive IMC. Their practical precision, however, is constrained by the capacitance contrast, thermal and circuit noise, parasitic capacitance, and the overhead of charge sensing and data conversion [33,34,35,36,37]. The access transistor in 1T–1C FeRAM provides cell selection and supports fast, mature array operation, but increases cell area and retains the restore overhead of destructive charge sensing [31,32]. A one-transistor FeFET offers intrinsic gain, compact non-volatile threshold storage, and direct compatibility with current-mode MAC, logic-in-memory, and associative search; its read margin and multilevel accuracy remain sensitive to interfacial traps, depolarization, threshold-voltage variation, and the coupling between program stress and endurance [39,43,44,45]. The separated FeMFET stack relaxes direct stress on the MOS gate dielectric and expands gate-stack design freedom, but requires careful capacitance-ratio matching and introduces additional area, interconnect, and floating-node complexity [61,62,63,64,65].

FTJs provide a dense two-terminal resistive element with non-destructive readout and analog or multilevel conductance modulation, which is well matched to passive crossbars and synaptic arrays. Their scalability is nevertheless limited by the uniformity and stability of the ultrathin barrier, low read current and sensing margin, device-to-device variability, nonlinear conductance updates, and array-level sneak paths [68,69,70,71,75]. Accordingly, no single topology is universally optimal: FeCAPs favor charge-domain accumulation with minimal static current; 1T–1C FeRAM favors selected, conventional non-volatile memory arrays; FeFETs and FeMFETs favor transistor-amplified current readout and logic integration, with FeMFETs offering additional stack decoupling; and FTJs favor dense resistive and neuromorphic arrays. Device selection should therefore be made jointly with the required read variable, update frequency, precision, array organization, and peripheral-circuit budget.

## 4. Application Progress of Hafnia-Based Ferroelectric Memories in In-Memory and Neuromorphic Computing

Section 4 examines how the same polarization-programmable HfO_2_ platform serves three application classes with distinct hardware objectives: high-throughput ANN/DNN inference, adaptive and event-driven neuromorphic processing, and sensor-side feature extraction. Although all three exploit non-volatile state storage and local computation, they do not impose equivalent device requirements. Inference emphasizes state separation and accumulation precision; learning emphasizes incremental-update fidelity and cycling endurance; and sensor-side processing emphasizes task-specific decision functions and the removal of data-conversion overhead.

### 4.1. Hafnia-Based Ferroelectric In-Memory Computing for ANN/DNN Acceleration

For ANN/DNN acceleration, the dominant kernel is VMM, for which programmed ferroelectric states represent weights and row voltages or pulse sequences represent inputs. Current- or charge-domain column accumulation then evaluates many products in parallel. The system-level benefit depends not only on reduced weight movement, but also on whether the required numerical precision can be obtained without allowing sensing and data-conversion overhead to offset the array-level energy gain.

**Figure 7:**
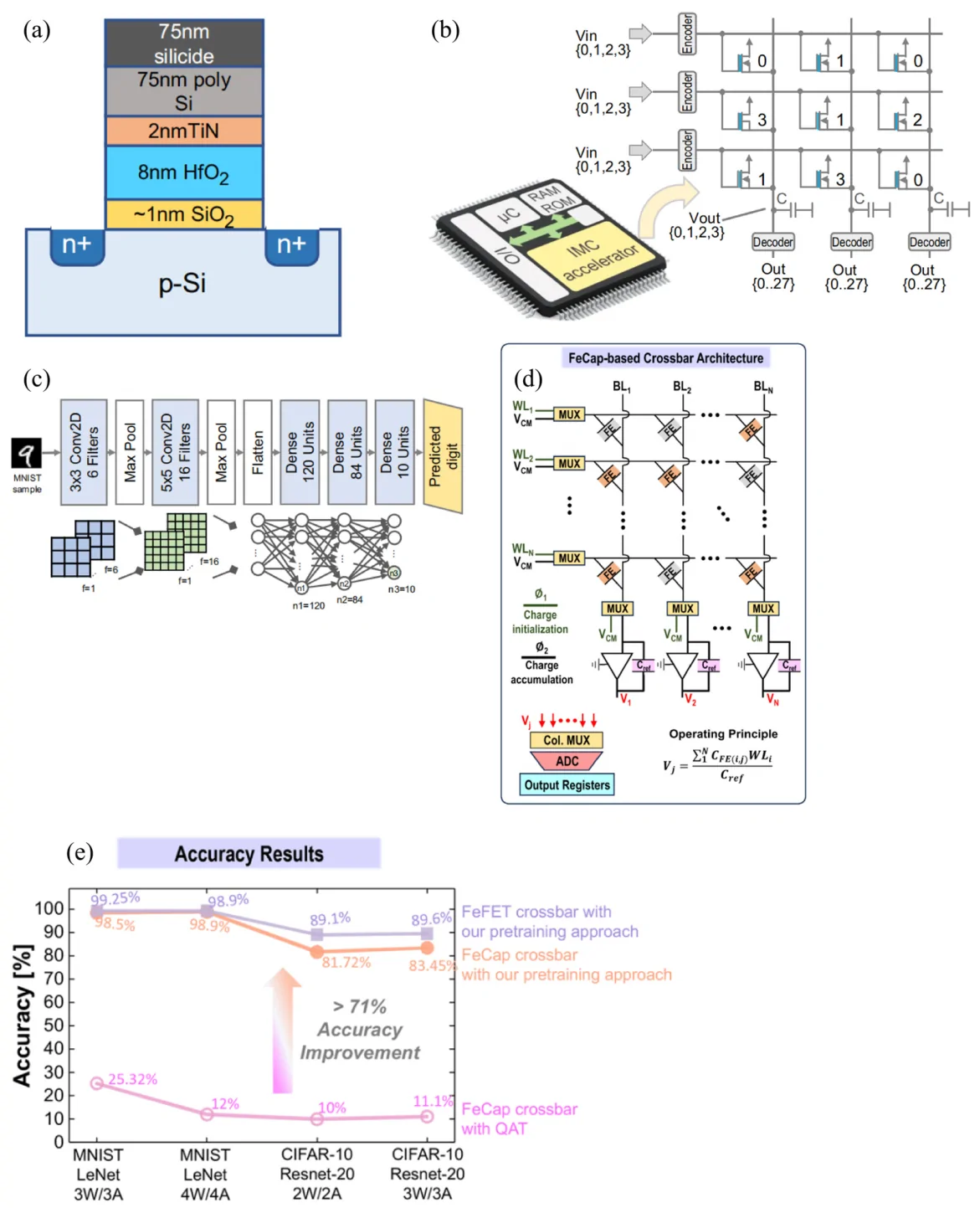
(**a**) The material stack of FeFETs. (**b**) IMC accelerators facilitate MAC operations for AI workloads, with the crossbar output accumulated as a capacitor voltage. (**c**) LeNet neural network tested for handwritten-digit recognition on the MNIST dataset, with all MAC layers quantized to 2 bits. Adapted from Ref. [78]. Copyright © 2023 The Author(s). Licensed under CC BY 4.0. (**d**) FeCAP-based IMC crossbar architecture for MVM computations. (**e**) Simulation results on the MNIST and CIFAR-10 datasets for LeNet and ResNet-20 models comprising FeCAP and FeFET crossbars. FeCAP crossbars based on quantization-aware training (QAT) exhibit lower accuracy, whereas the pretraining approach substantially improves the accuracy of the FeCAP-based neural network to a level comparable to the FeFET baseline. Here, QAT accounts for quantization but not FeCAP leakage. Adapted from Ref. [79]. Copyright © 2024 The Author(s). Licensed under CC BY 4.0.

Figure 7a,b illustrate the progression from the FeFET device stack to an array-level IMC architecture, providing a compact route toward non-volatile weight storage and in-array multiply–accumulate operations. In a FeFET, the polarization state of the HfO_2_-based ferroelectric gate stack modulates the threshold voltage of the underlying transistor. Partial polarization switching or controlled domain configurations enable multiple threshold-voltage states, which can be mapped onto discrete weight levels. The experimentally demonstrated multilevel FeFET crossbar architecture in Ref. [78] represents an important milestone by integrating multibit weight storage and MAC operation within a compact 1FeFET-1R cell. Rather than treating the FeFET as a binary memory element, this architecture exploits multiple threshold-voltage states for weight encoding and uses crossbar-level accumulation to implement neural-network-relevant computation.

The significance of this direction lies in the coexistence of non-volatility and logic-compatible integration. Compared with charge-based volatile memories, FeFETs can retain programmed weight states without static power consumption. Compared with many filamentary resistive memories, hafnia-based FeFETs are more naturally compatible with CMOS transistor processing and, in principle, can be integrated into advanced logic platforms. The multilevel FeFET crossbar illustrated in Figure 7b further shows that cell-level weight states can be translated into array-level MAC functionality, thereby bridging ferroelectric-device physics and system-level neural-network acceleration.

Beyond FeFETs, ferroelectric capacitors provide another important pathway for capacitive in-memory computing. As shown in Figure 7d, FeCAP-based arrays can use the remanent-polarization state as a non-volatile analog or multibit variable, while exploiting the capacitive response for charge-domain computation. Comparative studies of FeCAPs and FeFETs as machine-learning-oriented IMC elements indicate that the two device classes offer distinct but complementary advantages: FeFETs provide transistor-level readout gain and compatibility with current-mode crossbars, whereas FeCAPs offer compact two-terminal operation and potentially efficient charge-domain accumulation [79]. The cross-point HZO FeCAP architecture further broadens the scope of remanent-polarization-driven computation by demonstrating how polarization states can directly participate in array-level computing [80].

From an algorithmic perspective, the transition from memory cells to neural accelerators requires a careful mapping between physical device states and numerical weights. Figure 7c,e present representative neural-network demonstrations in which FeFET or FeCAP arrays are evaluated using standard image-recognition workloads. Such demonstrations are essential because device-level multistability alone does not ensure system-level accuracy. Ferroelectric-device non-idealities, including programming variation, nonlinear conductance update, limited state separation, retention loss, and read disturbance, must be incorporated into neural-network-level error models. Accordingly, recent studies have increasingly adopted device–circuit–algorithm co-design strategies, using bit slicing, mixed-signal accumulation, calibration, and algorithmic retraining to compensate for device non-idealities [81,82,83,84].

A key conceptual point is that HfO_2_-based ferroelectric IMC does not rely solely on the improvement of a single device metric. Instead, its performance is determined by the coordinated optimization of material switching characteristics, device programming schemes, array topology, peripheral circuits, and neural-network mapping. For ANN/DNN acceleration, the most relevant device-level attributes include multilevel programmability, state separation, low write energy, retention stability, endurance under repeated updates, and device-to-device uniformity. At the array level, parasitic resistance, capacitive loading, sneak paths, input/output precision, and analog-to-digital conversion overhead become equally critical. Therefore, the application progress summarized in Figure 7 should be interpreted as a gradual transition from individual ferroelectric memory demonstrations toward complete compute macros and workload-aware hardware evaluation.

Nevertheless, several challenges remain before hafnia-based ferroelectric IMC can be broadly deployed in high-density AI accelerators. First, the number of reliably distinguishable weight states is constrained by programming variability, noise, and readout margin. Second, analog MAC accuracy can be degraded by threshold-voltage drift, conductance variation, and peripheral-circuit mismatch. Third, although inference workloads can tolerate moderate device imperfections, on-chip training imposes more stringent requirements on update linearity, symmetry, and endurance. These issues motivate the system-level discussion in Section 4.4, where variation-aware learning and reliability engineering are examined as necessary bridges between device functionality and edge-AI deployment.

### 4.2. Hafnia-Based Ferroelectric Devices for Neuromorphic Computing

Neuromorphic computing uses the same programmable polarization states for a different objective: representing adaptive, history-dependent dynamics rather than only static coefficients for VMM. Gradual switching under tailored pulse sequences can provide analog synaptic updates, multiple memory timescales, and timing-dependent responses. Consequently, the relevant assessment shifts from MAC precision alone to update linearity, temporal fidelity, event energy, and the stability of learned states.

#### 4.2.1. Artificial Synapses and Multilevel Weight Modulation

Artificial synapses require non-volatile, analog, and preferably linear weight modulation. Hafnia-based ferroelectric devices can meet these requirements through several device-specific mechanisms. In FeFETs, ferroelectric polarization modulates the channel conductance or threshold voltage. In FTJs, polarization reversal changes the tunneling-barrier profile and thus the tunneling electroresistance. In FeCAP-based synapses, the remanent polarization or capacitance state can serve as a programmable weight. Across these device platforms, partial polarization switching enables intermediate states, which are essential for analog synaptic-weight representation.

**Figure 8:**
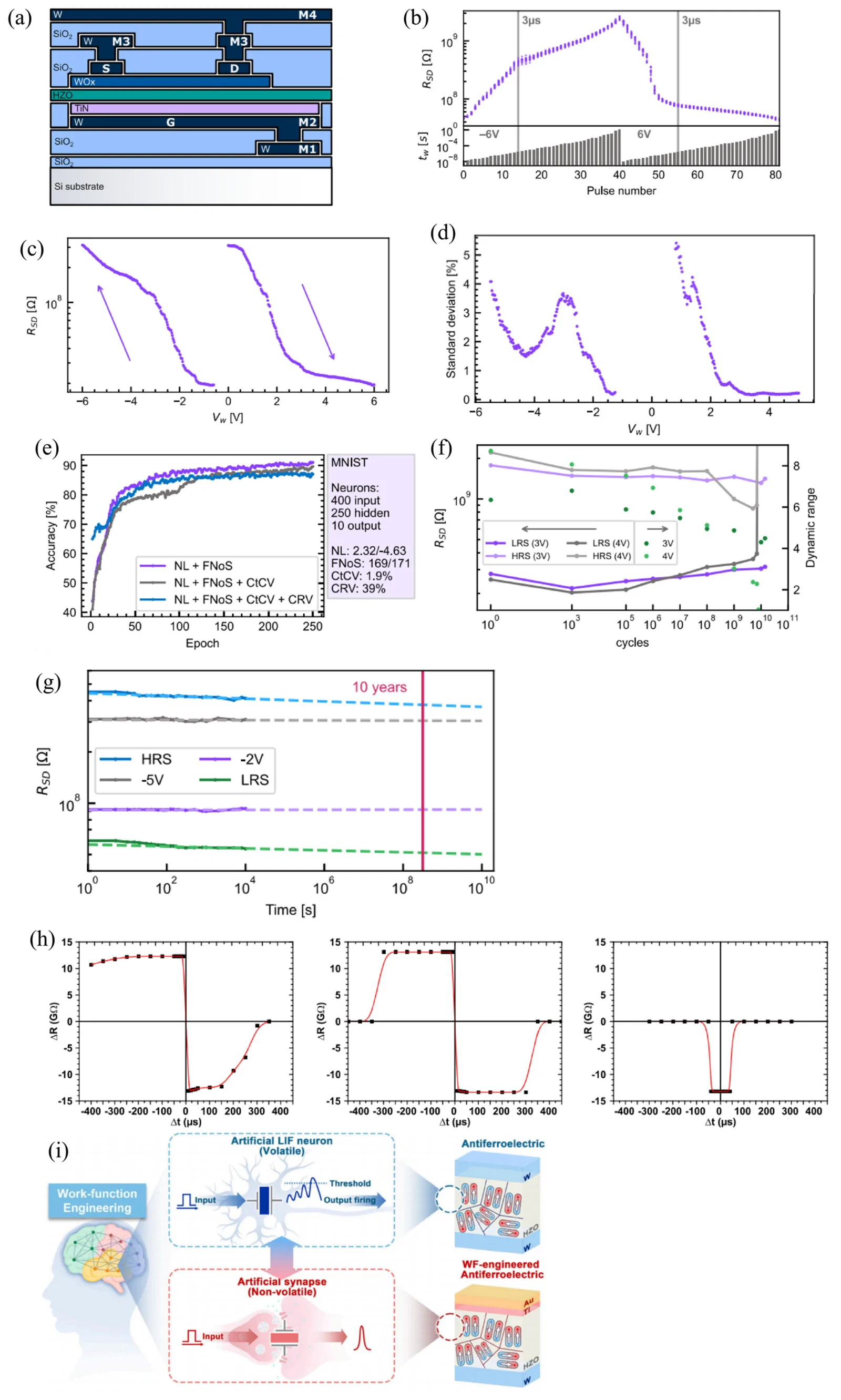
(**a**) Schematic illustration of the FeFET, indicating a source (S), a drain (D), a gate (G), a WOx channel, a ferroelectric HZO gate dielectric, G access metal line (M1, M2), S access metal line (M3), and D access metal line (M4). (**b**) Superposition as a function of the pulse number in each cycle to visualize the small cycle-to-cycle variations and potentiation and depression shape. The write pulse width tw was modulated from 10 ns to 1 s while keeping the write pulse amplitude Vw constant at 6 V (−6 V) for the potentiation (depression) on a FeFET with L = 400 nm and W = 1 μm. (**c**) One potentiation (0 V to 6 V) and depression (−0.6 V to −6 V) cycle with a constant tw = 500 μs showing quasi-continuous channel resistance states (dep: 241 states, pot: 217). The FeFET has a channel length of 300 nm and a channel width of 2 μm. (**d**) Standard deviation of a channel resistance state normalized by the resistance window (HRS-LRS) for each Vw. (**e**) MNIST classification performance of our FeFETs with different degrees of non-idealities included: nonlinearity factors and finite number of steps (purple), + cycle-to-cycle variation (gray), and+ conductance range variation (blue). (**f**) Endurance of a FeFET with L = 800 nm and W = 600 nm. Triangular pulses with a frequency of 100 kHz were applied up to 1010 cycles. The amplitude of the pulses was ±3 V and ±4 V. The evolution of the HRS and LRS (left axis) and the corresponding dynamic range (right axis) are shown. (**g**) Retention measurements at room temperature for a FeFET with L = 300 nm and W = 2 μm showing a good retention of >10 years for the four programmed states. Only the HRS has a small drift. The solid lines are the experimental data and the dashed lines are linear extrapolations in the log-log scale. Adapted from Ref. [85]. Copyright © 2023 The Author(s). Licensed under CC BY 4.0. (**h**) Prediction of the resulting STDP curve and the resistance change. Adapted with permission from Ref. [94]. Copyright © 2020 American Chemical Society. (**i**) Schematic diagram of the AFE HZO-based artificial neuron and synapse device by work function (WF) engineering. Adapted with permission from Ref. [95]. Copyright © 2024 American Chemical Society.

Figure 8a–c illustrate a representative HZO-based ferroelectric synaptic weight that combines ferroelectric field effects with additional channel–state modulation. The device structure in Figure 8a provides the physical platform, while Figure 8b,c show pulse-dependent potentiation/depression behavior and quasi-continuous resistance modulation. These results highlight an important advantage of hafnia-based ferroelectric synapses: the synaptic state can be tuned not only by pulse amplitude but also by pulse width, pulse number, and temporal programming history [85]. Such temporal programmability provides a richer design space than simple binary switching and enables more flexible control of analog synaptic weights.

Multilevel weight modulation in HfO_2_-based ferroelectrics is closely associated with multi-domain polarization dynamics. In thin HZO films, polarization reversal does not necessarily proceed as a single abrupt event; instead, partial switching of ferroelectric domains can produce intermediate polarization states. When these polarization states modulate a transistor channel or tunneling barrier, they produce graded conductance or resistance levels. This behavior is particularly valuable for neuromorphic hardware, where synaptic weights must be incrementally updated over many learning events. Studies on BEOL-compatible HZO FeFET synaptic weights, Al-doped HfO_2_ synaptic transistors, and HZO FTJ synapses collectively demonstrate that HfO_2_-based ferroelectrics can support programmable analog states across multiple device configurations [86,87,88,89].

However, multilevel behavior alone is insufficient for high-performance neuromorphic learning. The weight-update trajectory should ideally be linear, symmetric, reproducible, and energy-efficient. Figure 8d, which summarizes the cycle-to-cycle variability of an HZO-based synaptic device, highlights a central trade-off in ferroelectric synapse design: enhanced analog tunability is often accompanied by increased variability and reduced state precision. Consequently, recent efforts have moved beyond simply increasing the number of accessible states toward improving the quality of these states, including update linearity, temporal stability, and robustness under repeated programming [85,90,91].

#### 4.2.2. Synaptic Plasticity, Online/Offline Learning, and Neural-Network Task Demonstrations

Synaptic plasticity converts pulse history into a persistent or transient change in device state. In hafnia-based devices, this response can arise from the coupled evolution of ferroelectric domains, trapped charge, oxygen-vacancy distributions, and interface states; the useful quantity is therefore not simply the accessible state count, but the reproducibility with which a prescribed pulse sequence produces the intended weight change.

Figure 8e illustrates an online-learning demonstration based on experimentally extracted synaptic characteristics and neural-network simulation. Such results are important because they connect measured device dynamics with learning accuracy. In many neuromorphic demonstrations, the device is not used directly to train a large-scale network experimentally; instead, measured potentiation/depression curves, state noise, and variability are incorporated into simulation frameworks such as MLP or NeuroSim. This methodology enables evaluation of whether the measured synaptic characteristics are compatible with practical neural-network tasks [85,90,92].

A recurring challenge in ferroelectric synaptic learning is the balance between non-volatility and plasticity. Strong polarization retention is favorable for stable weight storage, whereas frequent learning requires reversible and reproducible state modulation. If the polarization state is excessively stable, incremental updates may require high voltages or become abrupt; if it is overly volatile, learned weights may decay over time. Multi-timescale HZO synaptic weights are therefore particularly relevant because they provide a route to emulate both transient and persistent memory components within the same device platform [85]. This dual-timescale behavior can be advantageous for online learning, adaptive inference, and reservoir computing, where memory fading and temporal response can serve as useful computational resources rather than detrimental non-idealities.

Task-level demonstrations further reveal the application relevance of hafnia-based ferroelectric synapses. HZO FTJ synapses have been evaluated for hardware neural-network applications and speech-recognition tasks, while HfO_2_-based ferroelectric neuromorphic devices with engineered insertion layers have been incorporated into reservoir-computing frameworks [76,89,92,93]. These studies indicate that HfO_2_-based ferroelectric devices are not limited only to static image classification but can also process temporal signals, where intrinsic device dynamics may actively contribute to computation. Such temporal-processing capability is particularly relevant for edge applications involving speech, biomedical signals, and sensor streams.

Reliability remains a central issue for learning-capable ferroelectric synapses. Figure 8f,g summarize the endurance and retention characteristics, respectively. Endurance determines whether the device can withstand repeated synaptic updates, whereas retention determines whether learned weights remain stable after programming. Although inference-only accelerators can tolerate a limited number of write cycles after offline training, online learning and continual adaptation impose substantially more stringent endurance requirements. Therefore, endurance and retention should not be regarded merely as peripheral memory metrics; rather, they directly define the feasible learning mode of the neuromorphic system.

#### 4.2.3. SNN, STDP, and Event-Driven Computing

Spiking neural networks (SNNs) provide a distinct neuromorphic computing paradigm in which information is encoded by the timing and frequency of discrete spikes rather than by continuous-valued activations. In such systems, synaptic devices are expected to respond to temporally correlated pre-synaptic and post-synaptic pulses. STDP is one of the most widely studied local learning rules, in which the synaptic weight update is governed by the relative timing between pre-synaptic and post-synaptic spikes.

Figure 8h presents a representative HZO-based FTJ demonstration of STDP. In this device class, tunneling electroresistance is modulated by the polarization state of the hafnia-based ferroelectric layer. By applying time-delayed voltage waveforms to the two electrodes, the effective voltage across the junction becomes dependent on spike timing, thereby producing timing-dependent conductance updates [94]. This mechanism is particularly appealing for SNN hardware because it provides a direct physical route to local synaptic learning without requiring global weight-update computation.

Beyond synaptic elements, event-driven neuromorphic systems also require neuron-like devices. Figure 8i illustrates an antiferroelectric HZO-based two-terminal synapse/neuron concept. Such devices extend the role of hafnia-based ferroelectrics from passive synaptic weights to active nonlinear elements capable of thresholding, excitability, and neuron-like switching [95]. FeFET-based oscillators and coupled-oscillatory networks further demonstrate that ferroelectric devices can participate in temporal and event-driven computation beyond conventional static weight storage [96,97]. These studies suggest that HZO-based ferroelectric devices can support multiple neuromorphic primitives, including synapses, neurons, oscillators, and reservoir nodes.

The distinction between ANN/DNN-oriented IMC and SNN-oriented neuromorphic computing is therefore not merely algorithmic; it also imposes fundamentally different device requirements. ANN/DNN accelerators prioritize high-density weight storage, linear MAC operation, and inference accuracy, whereas SNN systems require pulse responsiveness, local plasticity, temporal dynamics, and low event energy. HfO_2_-based ferroelectric devices are unusual in their potential to address both regimes: partial polarization switching enables analog weight storage, while pulse-dependent domain dynamics and tunneling modulation support temporal plasticity.

### 4.3. Sensing–Storage–Computing Integration and Sensor-Side Intelligent Processing

Sensor-side integration targets a different point in the dataflow: it performs task-relevant preprocessing before raw signals are transferred to a general-purpose processor. This placement is most valuable when the sensor produces high-dimensional data but the downstream task requires only sparse features, matches, or threshold decisions. Programmable ferroelectric states can retain local templates or decision parameters near the sensing front end, thereby replacing part of the conventional sensing–conversion–storage–processing chain rather than merely accelerating the same VMM kernel.

Low-power edge detection based on HfO_2_-based FeFETs provides a representative example of this concept. Conventional convolution-based edge-detection methods rely on repeated multiply–accumulate operations and analog-to-digital conversion, both of which contribute substantially to system energy consumption. In contrast, the matching univalue segment assimilating nucleus (MUSAN)-based strategy enables more localized feature extraction and matching by exploiting the non-volatile programmability of HfO_2_-based FeFETs. The corresponding array architecture supports local feature storage and comparison, allowing hardware-level edge-detection outputs to be generated with reduced data movement and computational overhead [98]. This task-specific implementation extends HfO_2_-based ferroelectric computing beyond generic matrix–vector multiplication toward dedicated perceptual-processing hardware.

The value of hafnia-based devices in this setting is determined by where computation is inserted into the sensor pipeline. Non-volatile threshold-voltage or conductance states can implement local matching, filtering, or thresholding, while CMOS-compatible integration can place these functions beside sensor readout and edge logic. Unlike a general ANN accelerator, however, a sensor-side macro should be judged primarily by the amount of data conversion and transmission it eliminates at a specified task accuracy, rather than by peak TOPS/W alone.

At the same time, sensing–storage–computing integration remains less mature than ANN/DNN-oriented IMC or artificial-synapse demonstrations. Many reported in-sensor computing platforms still rely on other material systems, including optoelectronic semiconductors, memristive oxides, and two-dimensional materials. Therefore, for hafnia-based ferroelectrics, the most rigorous current assessment is that FeFET-based local sensory processing has been experimentally validated in specific tasks such as edge detection, whereas broader HZO-based in-sensor computing systems remain at an emerging stage. Future work must further clarify how ferroelectric memory cells can be co-integrated with physical sensors, how analog sensory signals can directly modulate ferroelectric states, and how such systems can maintain accuracy under device variability and environmental perturbations.

### 4.4. Low-Power Edge Intelligence: System-Level Opportunities and Challenges

The applications above share a common device platform but expose different limiting metrics. ANN/DNN inference is primarily constrained by weight-state precision and peripheral conversion cost; online neuromorphic learning by update symmetry, endurance, and retention–plasticity balance; SNN processing by timing fidelity and event energy; and sensor-side processing by environmental stability and the extent of front-end data reduction. Accordingly, edge deployment should be assessed through application-specific error and energy budgets rather than through a single set of device figures of merit.

**Figure 9:**
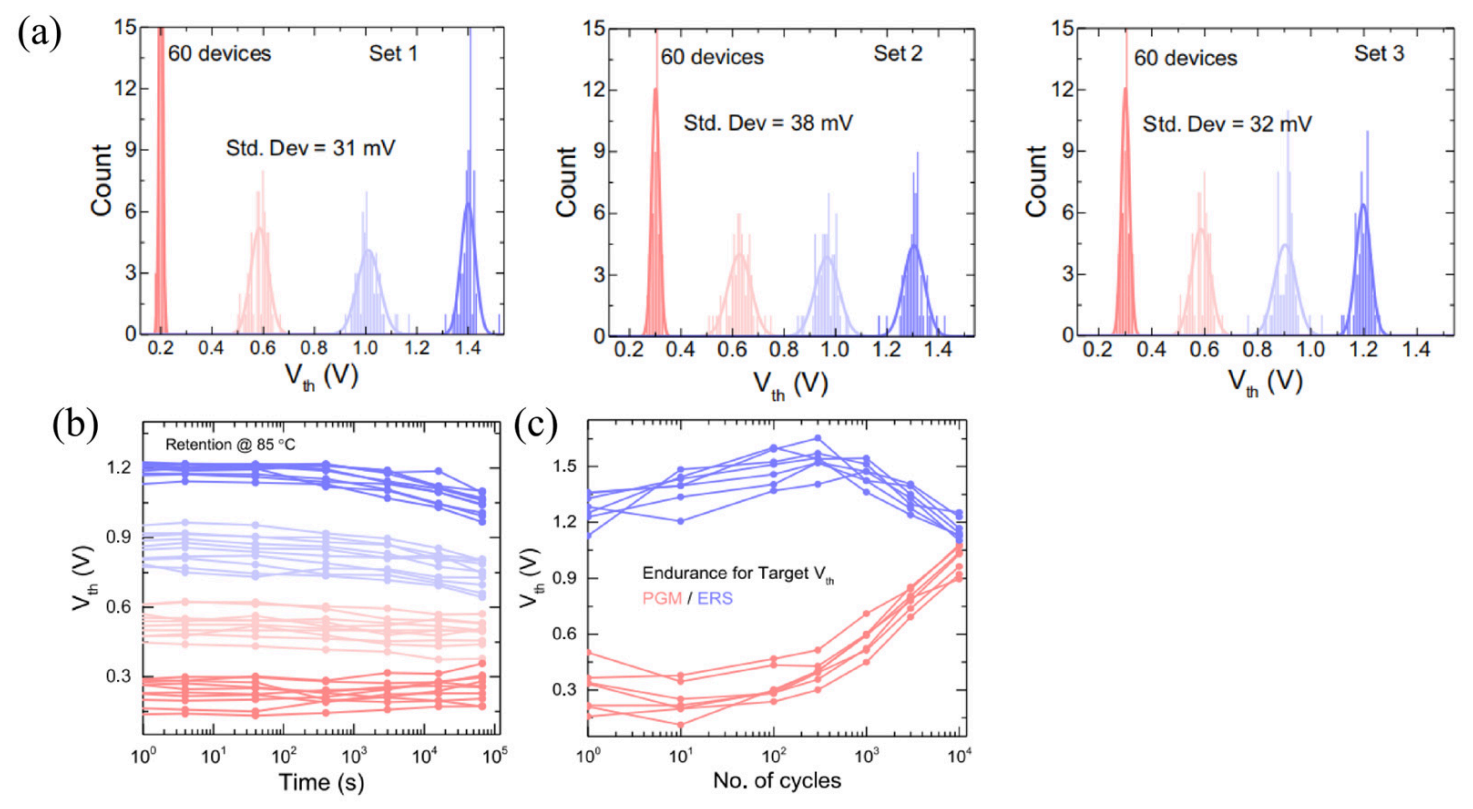
(**a**) Experimentally measured device-to-device variation with respect to Vth. (**b**) Experimentally measured retention of the four target Vth states at 85 °C over 24 h. (**c**) Experimentally measured endurance of the target Vth states over 10^4^ target program-erase cycles; each cycle comprises a standard erase followed by verified target programming using increasing pulse amplitudes, with a pulse width of 200 ns. Adapted from Ref. [78]. Copyright © 2023 The Author(s). Licensed under CC BY 4.0.

Figure 9a shows device-to-device Vth variation in multilevel FeFETs, which directly affects the mapping between programmed ferroelectric states and numerical weights. In small experimental arrays, such variation can be calibrated or compensated; in large-scale neural accelerators, however, statistical distributions of device states may propagate into inference errors. Figure 9b tracks the retention of the four target Vth states at 85 °C over 24 h, whereas Figure 9c shows the evolution of these states during repeated target program-erase cycling; the two panels therefore quantify temporal stability and update lifetime, respectively. These metrics are particularly important for edge systems, where recalibration may be infrequent and long-term autonomous operation is expected.

Variation is not only a device-reliability issue but also an application-dependent error source. In ANN/DNN inference, state dispersion perturbs numerical weights and accumulates across layers; in SNNs, it can alter firing thresholds and event timing; and in sensor-side processing, drift can shift local decision boundaries. Variation-aware Bayesian training demonstrates that measured FeFET uncertainty can be incorporated into the learning model instead of being treated solely as a materials defect [84]. This result supports a broader co-design principle: the acceptable variation envelope should be defined by task-level accuracy and recalibration cost, rather than by an application-independent device target.

Repeated switching imposes a similarly application-dependent reliability constraint. Fatigue, imprint, charge trapping, wake-up, and polarization relaxation can reduce the usable memory window and distort incremental updates [99,100]. Inference accelerators program weights relatively infrequently and can therefore tolerate a different endurance–retention balance from online-learning or adaptive-sensing systems, in which continual updates consume cycling lifetime. Device qualification should thus report stability under the write frequency, pulse protocol, and retention interval of the intended workload rather than extrapolating suitability from a single endurance value.

Application-level efficiency must likewise include the energy and latency of sensing, data conversion, calibration, error handling, and data movement. The FeFET edge-processing example in Ref. [98] shows that task-specific feature matching can avoid part of this overhead, but the same advantage cannot be assumed for a general mixed-signal accelerator. A device-level reduction in switching energy is systemically useful only when it reduces the dominant workload-level cost; if ADCs, verification, or communication dominate, circuit or dataflow changes will yield a larger benefit than further lowering the cell energy.

## 5. Challenges, Cross-Layer Limitations, and Outlook

**Figure 10:**
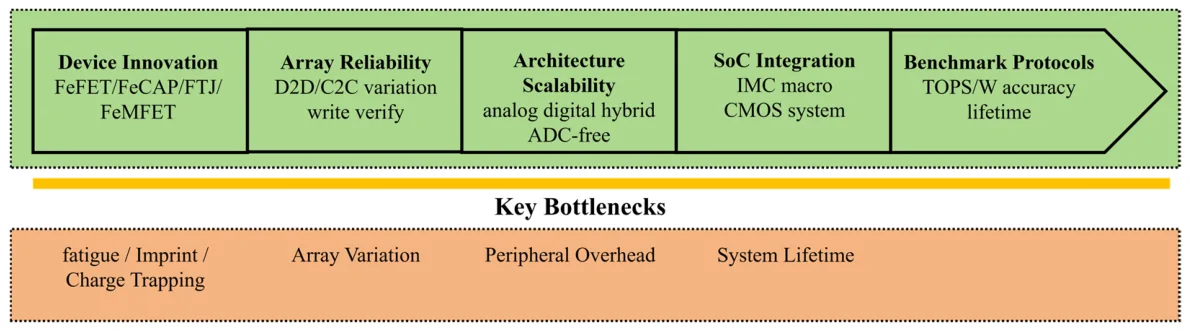
Roadmap toward reliable and scalable HZO-based in-memory computing.

Hafnia-based ferroelectric memories have evolved from a materials discovery into a broad hardware platform for memory-centric computing. Their attractiveness originates from a rare combination of non-volatility, CMOS compatibility, thickness scalability, and electrically programmable binary or multilevel states [1,2]. These features make HZO-based ferroelectric devices promising for IMC, neuromorphic computing, and edge-intelligent systems. Nevertheless, the central question is no longer whether hafnia-based ferroelectricity can be used for computation, but whether its device-level advantages can be preserved when translated into large arrays, peripheral circuits, architecture-level dataflows, and system-on-chip (SoC)-scale implementations. As illustrated in Figure 10, future HZO-based IMC should therefore be viewed as a cross-layer optimization problem rather than a device-only challenge. Materials and interface engineering determine the stability of ferroelectric switching; device platforms define the accessible memory states, readout mechanisms, and update dynamics; array reliability governs the fidelity of weight mapping and sensing margins; circuit and architecture design determines whether physical states can be exploited with sufficient precision and energy efficiency; and SoC-level integration and application-driven benchmarking ultimately determine whether the reported gains remain meaningful under realistic workloads.

### 5.1. Cross-Layer Bottlenecks in HZO-Based In-Memory Computing

Despite substantial progress, HZO-based IMC remains constrained by interdependent bottlenecks spanning materials, devices, arrays, circuits, and systems. At the materials level, wake-up, fatigue, imprint, depolarization, and charge trapping can alter the effective polarization state and induce memory-window drift [101,102]. These phenomena are not merely reliability concerns for stand-alone capacitors or transistors; in IMC arrays, they directly determine the long-term stability of stored weights and the reproducibility of repeated programming. For analog or multilevel computing, even small shifts in polarization, threshold voltage, capacitance, or tunneling resistance can lead to state overlap and accumulated computational error.

At the device level, FeFETs, FeCAPs, FTJs, FeMFETs, and antiferroelectric HZO devices provide distinct computing opportunities, while also introducing different trade-offs. Multilevel FeFET crossbars have demonstrated complete MAC operation using 28 nm high-k metal-gate FeFET devices, achieving 96.6% handwriting-recognition accuracy, 91.5% image-classification accuracy, and an energy efficiency of 885.4 TOPS/W [78]. Comparative studies of FeCAP and FeFET crossbars further indicate that FeCAPs can operate at low voltage and eliminate static current, whereas FeFETs provide a larger ON/OFF ratio and stronger readout margin [79]. These results suggest that device selection should be matched to the target computing mode rather than determined solely by memory density.

At the array level, variability remains a key obstacle. Device-to-device and cycle-to-cycle variations broaden the distribution of programmed states, reduce sensing margins, and degrade neural-network accuracy. In analog IMC, accumulated current or charge is further affected by parasitic resistance and capacitance, finite output precision, ADC/DAC overhead, and nonlinear update characteristics. Digital and ADC-free computing schemes can improve robustness, but often at the expense of additional logic complexity, area overhead, or bit-serial latency. Therefore, the practical bottleneck of HZO-based IMC lies not in a single material or device parameter, but in the propagation of non-idealities across the computing stack. Device-level performance metrics and circuit-level or application-level results are therefore summarized separately in Table 1 and Table 2, respectively.

### 5.2. Emerging Device Platforms for HZO-Based Computing

Device selection for HZO-based computing should be made against the transduction and peripheral requirements of the target workload. FeFETs combine non-volatile threshold states with transistor gain and are therefore well suited to current-mode MAC and digital or associative logic, but their usable precision is sensitive to threshold-voltage dispersion and read bias [78]. FeCAPs replace static read current with charge-domain accumulation and offer compact two-terminal cells, yet their modest capacitance contrast shifts the precision burden toward charge sensing and conversion [79]. Thus, the relevant comparison is not simply density or switching energy, but the joint cost of representing, reading, and accumulating each weight state.

FTJs offer a compact two-terminal path to analog conductance modulation and timing-dependent plasticity, whereas their low read current and conductance variability can tighten array-sensing requirements [94]. HZO/WO_x_ FeFET synapses demonstrate wide multilevel tuning and long retention, but online use must additionally satisfy update linearity and endurance requirements [85]. Antiferroelectric HZO devices can supply nonlinear synapse- or neuron-like responses [95], while FeMFETs separate the ferroelectric capacitor from the channel readout to reduce direct read-path stress at the cost of capacitance-ratio and floating-node design constraints. These trade-offs indicate that no universal platform dominates: inference, online learning, event-driven processing, and associative search favor different combinations of state precision, update dynamics, read margin, selector requirements, and peripheral complexity.

### 5.3. Variability-Tolerant Ferroelectric Arrays

Array reliability is one of the most important prerequisites for practical HZO-based IMC. In small device sets, variation can be manually calibrated or tolerated in proof-of-concept demonstrations. In large arrays, however, variation directly determines yield, accuracy, and lifetime. Device-to-device variability arises from film-thickness fluctuations, grain distribution, phase fraction, interface defects, dopant inhomogeneity, and local trap density. Cycle-to-cycle variability originates from stochastic domain switching, incomplete polarization reversal, charge trapping, and defect migration. These variations broaden the statistical distribution of weight states and make reliable multilevel operation increasingly difficult.

Reliability engineering has made substantial progress at the materials and capacitor levels. Engineered recovery protocols in HZO capacitors can restore (2P_r_) to values above 40 μC/cm^2^ and extend endurance beyond 10^9^ cycles [99]. At the array level, however, high endurance alone is insufficient; statistically stable multilevel states must also be maintained under repeated programming and readout.

A future HZO-based IMC array should therefore be designed as a variability-tolerant system rather than an ideal analog memory. Write-verify schemes can reduce programming errors but inevitably increase latency and energy consumption. Closed-loop programming improves state precision, but the associated peripheral cost must be amortized over sufficiently large arrays. Redundancy and differential encoding enhance robustness but reduce effective storage density. Calibration and retraining can recover inference accuracy but require compact variation models and runtime support. Variation-aware learning therefore provides a particularly promising path. Experimental measurements of 28 nm FeFETs show that cycle-to-cycle and device-to-device conductance variations are state-dependent, size-dependent, and read-voltage-dependent. When these measured variation statistics are incorporated into Bayesian neural-network training, shallow MNIST networks retain near-ideal accuracy, while deeper AlexNet/CIFAR-10 networks exhibit only limited accuracy degradation of approximately 3.8–16.1% over a broad range of variation conditions [84]. This result suggests that complete elimination of variability may not be necessary; instead, the objective should be to co-design arrays and algorithms such that the remaining variability becomes tolerable for the target workload.

### 5.4. Precision-Scalable IMC Architectures

The architecture of HZO-based IMC must reconcile two competing objectives: high energy efficiency and sufficient numerical precision. Analog IMC offers massive parallelism by exploiting physical current-domain or charge-domain accumulation, but its precision is limited by device variation, line resistance, nonlinear update behavior, ADC/DAC quantization, and peripheral mismatch. Digital IMC provides more deterministic operation and stronger compatibility with verification flows, but often requires additional transistors, logic stages, or bit-serial operation. Hybrid architectures seek to balance these trade-offs by combining analog accumulation with digital correction, bit slicing, time-domain encoding, or ADC-free logic.

Precision scalability should therefore become a central design principle. Rather than targeting a fixed precision across all workloads, HZO-based IMC architectures should support task-dependent precision allocation. Low-precision inference, binary neural networks, ternary networks, and associative search may benefit from digital or ADC-free FeFET logic. Higher-precision DNN inference may require bit-sliced FeFET or FeCAP arrays with calibrated accumulation. Online learning and neuromorphic computing may rely on analog weight modulation but only when update nonlinearity and asymmetry are explicitly incorporated into the training algorithm.

Peripheral circuits are decisive in this comparison. Although a device may exhibit low switching energy, system-level energy can be dominated by ADCs, DACs, sense amplifiers, level shifters, write drivers, and calibration circuits. Benchmarking studies comparing analog and digital IMC emphasize that the two paradigms occupy distinct design spaces in terms of accuracy, efficiency, and dataflow flexibility [104]. Moreover, device–circuit–architecture co-exploration frameworks show that variation-aware design can substantially improve robustness, reducing accuracy loss under device variation from 76.44% to 0.45% while achieving an energy efficiency of 16.3 TOPS/W [103]. These studies suggest that HZO-based IMC should not be evaluated by peak cell-level or macrolevel metrics alone, but by workload-aware energy, latency, accuracy and precision scaling.

### 5.5. SoC Integration of HZO-Based IMC Macros

The ultimate impact of HZO-based IMC will depend on whether ferroelectric computing macros can be integrated into complete SoCs. In practical systems, an HZO IMC macro will coexist with CMOS logic, SRAM caches, non-volatile storage, sensors, interconnects, power-management circuits, and software-controlled dataflows. Therefore, the relevant question is not only whether an HZO device can perform computation but also where the HZO macro should be positioned within the memory hierarchy and which workloads should be mapped onto it.

Several integration scenarios are possible. HZO-based IMC can function as an edge-AI coprocessor for low-power inference, a near-memory accelerator for matrix operations, a content-addressable memory for search and pattern matching, or a local processing block adjacent to sensors. The FeFET-based MUSAN edge detector provides an instructive example: by mapping ADC-free feature matching onto a 4 × 4 FeFET NAND array, the system achieves approximately 10 fJ per operation and hardware edge detection without accuracy loss [98]. This illustrates that task-specific computing can relax analog precision requirements while substantially reducing peripheral overhead.

SoC integration also introduces constraints that are often absent from device-level demonstrations. These constraints include power delivery, thermal budget, packaging, testability, yield, programming interfaces, error monitoring, and compiler/runtime support. BEOL compatibility is a major advantage of hafnia-based ferroelectrics, but BEOL integration also imposes thermal and process restrictions. Peripheral-circuit amortization therefore becomes critical: although a small IMC array may exhibit impressive cell-level energy, a system-level macro must distribute the cost of sensing, programming, control, and data movement over sufficient useful compute density. Future demonstrations should therefore move beyond isolated arrays toward programmable macros with realistic interfaces, workload mappings, and system-level evaluation.

### 5.6. Benchmarking Protocols for Application-Level Evaluation

Standardized benchmarking is essential for the fair evaluation of HZO-based IMC. Current studies report a wide range of metrics, including switching energy, memory window, endurance, retention, number of programmable states, MAC energy, TOPS/W, inference accuracy, and array size. However, these metrics are often extracted under different assumptions and therefore cannot be directly compared. Cell-level switching energy measured using ideal voltage pulses does not represent the total energy of an accelerator, which also includes programming, sensing, conversion, control, and data movement. Similarly, high endurance measured in a capacitor does not automatically translate into reliable online learning in an array, where multilevel-state stability, update statistics, read disturbance, and peripheral feedback must also be considered.

Future benchmarking should therefore be application-driven. For ANN/DNN inference, essential metrics should include array size, weight precision, input precision, ADC/DAC resolution, TOPS/W, TOPS/mm^2^, latency, peripheral energy ratio, and accuracy degradation relative to software baselines. For online learning, endurance-aware accuracy, update linearity, update asymmetry, cycle-to-cycle variation, and retraining cost should be reported. For SNN and neuromorphic systems, event energy, temporal response, STDP fidelity, retention time constants, and spike sparsity are more relevant than conventional MAC-centric metrics. For sensing–storage–computing systems, benchmarks should further include sensor-interface energy, ADC reduction, preprocessing accuracy, and task-level latency. Such application-specific benchmarking would make it possible to distinguish genuine system-level advantages from isolated device-level improvements.

The roadmap in Figure 10 emphasizes that benchmarking should not be treated merely as a final reporting step after device and architecture design. Instead, it should provide feedback to earlier design stages. If a target application requires only binary or ternary weights, device research should prioritize reliability and density rather than unnecessary analog precision. If a workload requires online adaptation, endurance and update symmetry become central design targets. If peripheral energy dominates the system budget, architectural innovation may be more impactful than marginal reductions in switching voltage. Quantitative IMC benchmarking frameworks have already shown that peak macrolevel metrics cannot replace full-system workload evaluation, reinforcing the need for workload-aware modeling and standardized reporting [105].

## 6. Conclusions

Ferroelectric hafnium oxide has brought ferroelectric computing into a regime that was difficult to access with conventional perovskite materials: nanoscale thickness, CMOS-compatible processing, non-volatility, and field-driven switching can now coexist within a semiconductor-relevant material platform. This combination makes HZO-based ferroelectric memories more than a new class of storage devices. Their polarization states, switching dynamics, and history-dependent electrical responses provide physical degrees of freedom that can be directly exploited for local computation, neural-network acceleration, associative search, synaptic weighting, and sensor-side intelligence.

The development of HZO-based in-memory computing is fundamentally governed by cross-layer interactions spanning materials, devices, arrays, circuits, and systems, rather than by isolated improvements in individual performance metrics. Material engineering determines polarization stability, defect evolution, and long-term reliability; device design defines the mechanisms for state programming, readout, and retention; array-level implementation introduces variability, parasitic effects, and statistical uncertainty; circuit architecture dictates computational precision, throughput, and energy efficiency; and system-level integration ultimately determines whether the intrinsic advantages of ferroelectric devices can be translated into practical gains under realistic application workloads. Recent advances have demonstrated the feasibility of exploiting ferroelectric switching for computation beyond conventional data storage, highlighting the emergence of HZO-based memories as active computing elements. At the same time, these developments underscore that reliability degradation, device variability, and peripheral-circuit overhead remain interdependent challenges that must be addressed through coordinated optimization across the computing stack.

Future progress should therefore move beyond the search for an ideal ferroelectric device and toward application-defined co-design. For inference-oriented accelerators, stable multilevel states, readout margin, and peripheral efficiency are critical. For online learning and neuromorphic systems, update symmetry, endurance, retention dynamics, and temporal response become equally important. For edge-intelligent systems, task-specific architectures that avoid unnecessary analog precision or ADC overhead may provide a more practical route than pursuing universal computing macros. In this context, device non-idealities should not only be suppressed at the materials level but also modeled, calibrated, and compensated at the array, circuit, and algorithm levels.

Overall, HZO-based ferroelectric memories are positioned at the intersection of materials innovation and memory-centric computing architecture. Their future impact will depend on whether the field can establish a unified design methodology that links ferroelectric stack optimization, variability-tolerant arrays, precision-scalable architectures, and SoC-level benchmarking. If this cross-layer transition is achieved, hafnia-based ferroelectric technology could become a key enabler for energy-efficient computing systems in which storage, logic, learning, and perception are no longer physically and functionally separated.

## Figures and Tables

**Table 1:** Representative device-level performance metrics of HZO-based ferroelectric platforms.

Device Platform	Evaluated Metric(s)	Representative Value(s)	Device-Level Implication	Refs.
Interface-engineered HZO FeCAP	Fatigue-free switching	>10^11^ cycles	Interface design suppresses fatigue	[22]
Interface-engineered HZO FeCAP	Projected endurance lifetime	>10^12^ cycles	Oxygen-vacancy regulation is critical	[22]
HZO FeCAP with recovery protocol	Recovered polarization	2Pr > 40 μC/cm^2^	Fatigue recovery can restore switching	[99]
HZO FeCAP with recovery protocol	Recovery endurance	>10^9^ cycles	Online-learning endurance remains demanding	[99]
FeFET	ON/OFF ratio	4.9 × 10^6^	Large current contrast provides a strong read margin	[79]
FeCAP	Capacitance ratio (Cratio)	1.2–1.29	Modest capacitance contrast limits charge-domain state separation	[79]
HZO/WO_x_ FeFET synaptic device	Resistance tuning	~60×	Large analog weight window	[85]
HZO/WO_x_ FeFET synaptic device	Programmable states	>200 states	Multilevel synaptic programmability	[85]
HZO/WO_x_ FeFET synaptic device	Endurance/retention	>10^10^ cycles/>10 years	Long-term synaptic stability	[85]

**Table 2:** Representative circuit- and application-level performance metrics of HZO-based in-memory computing.

Implementation/Application	Evaluated Metric(s)	Representative Value(s)	Evaluation Context	Ref.
Multilevel FeFET IMC	Energy efficiency	885.4 TOPS/W	Multilevel FeFETs enable efficient MAC	[78]
Multilevel FeFET IMC	Task accuracy	96.6%/91.5%	Accuracy depends on multilevel-state control	[78]
Variation-aware neural-network modeling	CIFAR-10 accuracy degradation	~3.8–16.1%	Accuracy loss depends on network depth and measured device-variation conditions	[84]
Device–circuit–architecture co-exploration	Accuracy loss under variation	0.45% vs. 76.44%	Co-exploration suppresses variation impact	[103]
Device–circuit–architecture co-exploration	Energy efficiency	16.3 TOPS/W	Cross-layer architecture search improves efficiency	[103]
FeFET-based edge-processing system	Energy per operation	~10 fJ/op	ADC-free task-specific IMC is promising	[98]
FeFET-based edge-processing system	Hardware agreement	100% on 200 images	Feature matching improves robustness	[98]

## Data Availability

Data are contained within the article.
